# On the Reaction of 1,3-Diphenylisobenzofuran and (2-Iodoethynyl)(phenyl)iodonium Triflate. A Unique Case of Oxygen Transfer from the Diels-Alder Adduct to the Diene ^†^

**DOI:** 10.3390/molecules17088795

**Published:** 2012-07-25

**Authors:** Pelayo Camps, Tània Gómez, David Lozano, Teresa Calvet, Mercè Font-Bardia

**Affiliations:** 1 Pharmaceutical Chemistry Laboratory (CSIC Associated Unity), Faculty of Pharmacy, Biomedicine Institute of the University of Barcelona (IBUB), University of Barcelona, Joan XXIII av., Barcelona E-08028, Spain; Email: tgomezna7@alumnes.ub.edu (T.G.); lozanme7@alumnes.ub.edu (D.L.); 2 Crystallography, Mineralogy and Mineral Deposits, University of Barcelona, Martí Franquès st., Barcelona E-08028, Spain; Email: mtcalvet@ub.edu (T.C.); mercefont@ub.edu (M.F.-B.); 3 X-ray Diffraction Unity, Scientific and Technologic Centre of the University of Barcelona (CCiTUB), University of Barcelona, Solé i Sabarís st. 1–3, Barcelona E-08028, Spain

**Keywords:** NMR spectroscopy, structure elucidation, X-ray diffraction

## Abstract

Reaction of 1,3-diphenylisobenzofuran (DPIBF) with 2-(iodoethynyl)(phenyl)-iodonium triflate at room temperature gave the expected Diels-Alder adduct, but using an excess of DFIBF (2 equiv.) and performing the reaction at 55 °C or heating at this temperature during the concentration stage, the initial orange solution or product mixture became dark brown and the products 1,2-phenylene-1,2-bis(phenylmethanone) and 2-(3-iodo-1,4-diphenylnaphthyl)(phenyl)iodonium triflate were obtained, which suggests an oxygen transfer between DPIBF and the initial adduct.

## 1. Introduction

Some time ago, Stang *et al.* described the preparation of β-functionalized alkynyl(phenyl)iodonium triflates and 1,2-bis[[(phenyl)trifluoromethanesulfonyloxy]iodo]acetylene and their Diels-Alder reactions with different dienes, such as cyclopentadiene, 1,3-cyclohexadiene, 2,3-dimethylbutadiene, furan and 1,3-diphenylisobenzofuran (DPIBF, **2**) [1,2]. They also described the reaction of these adducts with different nucleophiles to give the corresponding substitution products [3,4]. Recently, we described the preparation of (2-iodoethynyl)(phenyl)iodonium triflate (**1**) and its reaction with a 1,1-disubstituted cyclopentadienes to give the expected Diels-Alder adducts [5]. Herein, we describe an unexpected result observed in the reaction of triflate **1** with diene **2**.

## 2. Results and Discussion

**Scheme 1:**
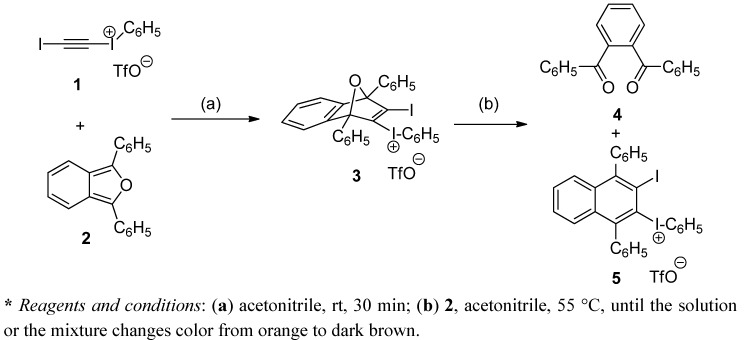
Reaction of 2-(iodoethynyl)(phenyl)iodonium triflate (**1**) with DPIBF (**2**).

Triflate **1** was reacted with diene **2** (2 equiv.) in acetonitrile solution at room temperature for 20 h, and the orange reaction mixture was concentrated in a rotary evaporator at 55 °C, taking at a certain moment a dark brown color. Upon crystallization from CH_2_Cl_2_/Et_2_O, a mixture containing 1,2-phenylene-1,2-bis(phenylmethanone) (**4**) was obtained, diene **2** being not observed. Crystallization of the above mixture from CH_2_Cl_2_/toluene (twice) gave (3-iodonaphthalen-2-yl)(phenyl)iodonium triflate (**5**) in 54% yield, instead of the expected Diels-Alder adduct (**3**) (Scheme 1).

**Figure 1:**
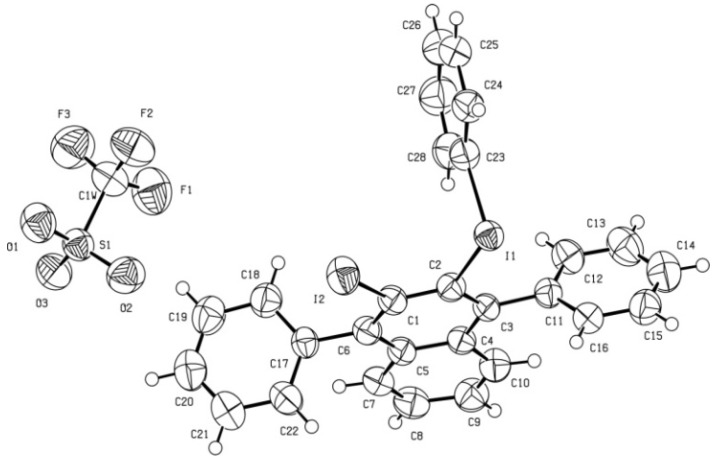
.ORTEP of triflate **5**.

**Scheme 2:**
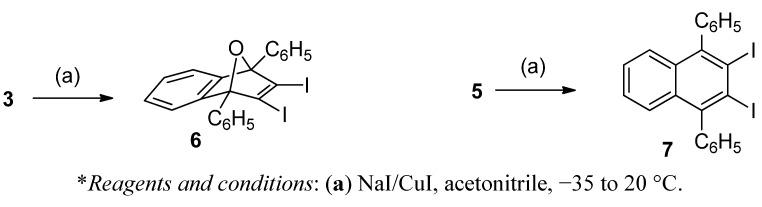
Conversion of triflates **3** and **5** into diiodides **6** and **7**.

The ^1^H- and ^13^C-NMR data and the elemental analysis did not allow us to differentiate among triflates **3** and **5**. However, the accurate mass measurement of the obtained product showed the molecular ion to have an *m/z* value (608.9567 amu) 16 units less than expected for the cation of triflate **3**. This molecular ion might correspond to the cation of triflate **5**. The structure of **5** was subsequently clearly established by X-ray diffraction analysis (Figure 1). Reaction of triflate **5** with NaI and CuI in acetonitrile [3] gave the recently described [6] 2,3-diiodo-1,4-diphenylnaphthalene (**7**), which was fully characterized (Scheme 2).

**Figure 2:**
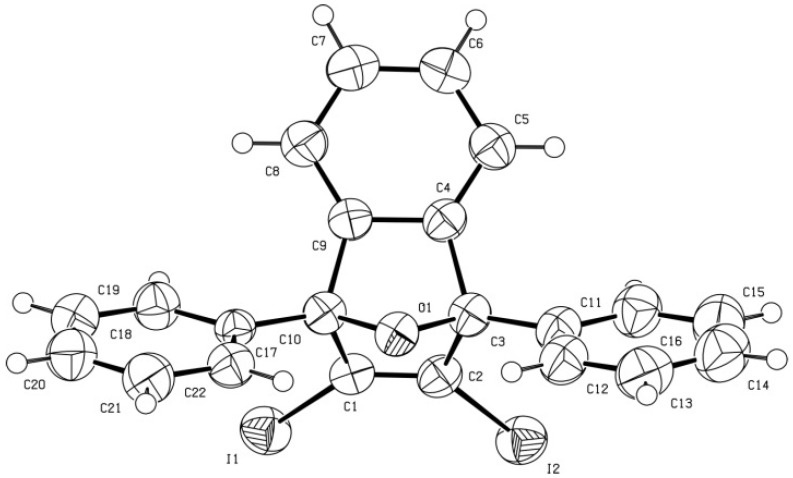
ORTEP of diiodide **6**.

When the reaction of diene **2** (2 equiv.) and triflate **1** was carried out at room temperature for 24 h and the solvent was evaporated under reduced pressure without heating, a residue containing much starting diene **2** was obtained. Crystallization of this residue from CH_2_Cl_2_/Et_2_O, always at room temperature, gave triflate **3** in 67% yield. This triflate was not too stable, and at room temperature the light yellow solid initially obtained became dark within 24 h. It also decomposed on drying *in vacuo* at room temperature. However, it could be characterized by spectroscopic means (^1^H-, ^13^C- and ^19^F-NMR spectra and accurate mass measurement). The ^1^H- and ^13^C-NMR spectra could be completely assigned on the basis of ^1^H/^1^H homocorrelation (COSY ^1^H/^1^H) and ^1^H/^13^C heterocorrelation spectra (HSQC and HMBC sequences). Moreover, triflate **3**, on reaction with NaI/CuI in acetonitrile (Scheme 2), was transformed in high yield into the new diiodide **6**, whose structure was fully established on the basis of its analytical and spectroscopic data, including an X-ray diffraction analysis (Figure 2).

To establish the conditions under which triflates **3** or **5** are formed, we performed the reaction of diene **2** and triflate **1** under different conditions, always in acetonitrile. Thus, reaction of diene **2** (1.2 equiv.) and triflate **1** at room temperature for 30 min gave an orange solution. After concentration and crystallization of the orange residue from CH_2_Cl_2_/Et_2_O, without heating at any moment, triflate **3** was obtained in 92% yield. A similar reaction performed for 20 h gave triflate **3** in quantitative yield. Reaction of diene **2** (2 equiv.) and triflate **1** at 55 °C for 24 h gave a dark brown solution. After concentration and crystallization from CH_2_Cl_2_/Et_2_O and CH_2_Cl_2_/toluene (twice), always at room temperature, triflate **5** was obtained in 42% yield. When diene **2** (2 equiv.) and triflate **1** were reacted at room temperature for 30 min an orange solution was obtained, which on concentration in a rotary evaporator heating at 55 °C gave a dark brown residue. After crystallization as before, triflate **5** was obtained in 42% yield.

An attempt to reduce triflate **3** to **5** by reaction with an excess of dimethylsulfide in CH_2_Cl_2_ at room temperature left the starting triflate **3** unchanged. When the above reaction was carried out in a closed vessel at 55 °C, complete degradation of triflate **3**, but not formation of triflate **5** was observed. A similar result was obtained on heating a solution of triflate **3** in acetonitrile at 55 °C for 3 h.

Consequently, it appears that triflate **3** is unstable and decomposes at temperatures around 55 °C. Decomposition of triflate **3** in the presence of DPIBF takes place in part by oxygen transfer between them. This transformation is clearly observed by a change in the color of the reaction mixture (solution or concentrate) from orange to dark brown.

**Scheme 3:**
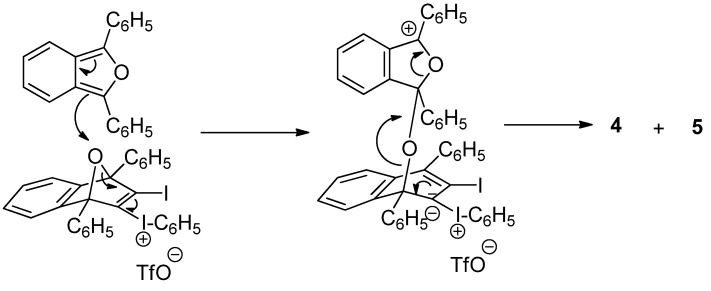
Possible mechanism for the conversion of triflate **3** to **5** on reaction with DPIBF (**2**).

A possible mechanism for the reaction of DPIBF with triflate **3** to give dione **4** and triflate **5** is given in Scheme 3, which starts with the electrophilic attack of the cation of triflate **3** on the electron-rich diene **2**. Although DPIBF can be oxidized by air [7], it seems clear that in our case, most of dione **4** comes from the oxidation of DPIBF by adduct **3**. As previously indicated, much diene **2** is observed in the reaction of triflate **1** with excess of DPIBF, when adduct **3** is the main product. Conversely, dione **4** but no diene **2** is the main by-product from the reaction of triflate **1** with an excess of DPIBF, if adduct **3** has been transformed into triflate **5**.

## 3. Experimental

### 3.1. General

Melting points were determined in open capillary tubes. Unless otherwise stated, NMR spectra were recorded at 25 °C in CDCl_3_ solution: ^1^H-NMR (400 MHz), ^13^C-NMR (100.6 MHz), ^19^F (376.29 MHz). All chemical shifts (δ_H_, δ_C_ and δ_F_) are reported in parts per million (ppm) related to internal standards (CHCl_3_ at δ_H_ = 7.26 ppm, δ_C_ = 77.0 ppm and CF_3_COOH at δ_F_ −76.55 ppm). Assignments given for the ^1^H and ^13^C-NMR spectra are based on DEPT sequences, ^1^H/^1^H COSY, ^1^H/^13^C HETCOR (gHSQC and gHMBC sequences for one bond and long range correlations, respectively). In the case of triflate **3**, previous assignment for the adduct of methyl 1-benzylcyclopenta-2,4-dienecarboxylate and triflate **1** were taken into account [5]. Coupling constants *J* are given in Hertz (Hz). Mass spectra were recorded on an LC/MSD-TOF instrunent (2006, Agilent Technologies), using electrospray (ESI-MS, positive mode, capillary: 3.5 kV, fragmentor: 215 V). Unless otherwise stated, IR spectra were recorded using the attenuated total reflection (ATR) technique and the absorption values are given as wavenumbers (cm^–1^). Elemental analyses were done at the Microanalysis Service of the Institut de Química Avançada de Catalunya (IQAC, CSIC, Barcelona, Spain). Column chromatography was performed on silica gel 60 A C.C. (35–70 mesh). For the thin layer chromatography (TLC), aluminum-backed sheets with silica gel 60 F254 were used and spots were visualized with UV light and/or 1% aqueous KMnO_4_.

*(3-Iodo-1,4-diphenyl-1,4-dihydro-1,4-epoxynaphthalen-2-yl)(phenyl)iodonium triflate* (**3**). To a magnetically stirred suspension of DPIBF (**2**, 150 mg, 0.55 mmol) in anhydrous acetonitrile (2.5 mL), a solution of triflate **1** (233 mg, 0.46 mmol) in anhydrous acetonitrile (1 mL) was added dropwise under an Ar atmosphere and the mixture was vigorously stirred at room temperature for 20 h. The orange solution was concentrated *in vacuo* at room temperature and the orange solid residue (418 mg) was treated with CH_2_Cl_2_/Et_2_O (1:3, 2 mL). The solid triflate **3** (356 mg, quantitative yield) was isolated as a light yellow solid, m.p. 80–83 °C (dec.) by decantation and washing with Et_2_O (3 × 0.5 mL) and CH_2_Cl_2_ (3 × 0.5 mL). On drying *in vacuo* at room temperature this salt became black. The salt was stable for weeks at 4 °C, but decomposed after standing at room temperature for one day. The analytical sample was not fully dried and contained traces of Et_2_O and CH_2_Cl_2_. IR (ATR) ν: 3061, 1548, 1494, 1472, 1446, 1287, 1228, 1220, 1180, 1166, 1156, 1021, 993, 916, 905, 851, 835, 757, 737, 703, 675, 653, 633, 591 cm^−1^. ^1^H-NMR δ: 7.10 (dt, *J* = 2.0 Hz, *J′* = 9.0 Hz, 1H, 7-H), 7.15 (dt, *J* = 2.0 Hz, *J′* = 9.0 Hz, 1H, 6-H), 7.19 (tm, *J* = 9.5 Hz, 2H, Ar-H_meta_Ar-I^+^), 7.30 (ddm, *J* = 8.8 Hz, *J′* = 1.5 Hz, 2H, Ar-H_ortho_ArI^+^), 7.38 (dd, *J* = 9.0 Hz, *J′* = 1.5 Hz, 1H, 8-H), 7.48 (tm, *J* = 9.0 Hz, 1H, Ar-H_para_ArI^+^), 7.50–7.60 (complex signal, 7H, 5-H, C1-Ar-H_meta_, C4-Ar-H_meta_, C1-Ar-H_para_ and C4-Ar-H_para_), 7.75 (dm, *J* = 8.0 Hz, 2H, C1-Ar-H_ortho_), 7.85 (dm, *J* = 7.5 Hz, 2H, C4-Ar-H_ortho_). ^13^C-NMR δ: 96.3 (C, C1), 97.6 (C, C4), 115.1 (C, C2-I^+^-Ar-C_ipso_), 120.0 (C, q, *J* = 320 Hz, CF_3_SO_3_), 121.8 (CH, C8), 122.4 (CH, C5), 126.4 (CH, C6), 126.5 (CH, C1-Ar-C_ortho_), 126.9 (CH, C7), 127.4 (CH, C4-Ar-C_ortho_ and C, C1-Ar-C_ipso_), 128.5 (CH) and 129.3 (CH) (C1-Ar-C_meta_ and C4-Ar-C_meta_), 129.6 (CH, C4-Ar-C_para_), 129.8 (CH, C1-Ar-C_para_), 132.0 (CH, C2-I^+^-Ar-C_meta_), 132.3 (CH, C2-I^+^-Ar-C_para_), 132.7 (C, C4-Ar-C_ipso_), 134.3 (CH, C2-I^+^-Ar-C_ortho_), 140.6 (C, C3), 142.2 (C, C2), 145.8 (C, C4a), 146.4 (C, C8a). ^19^F-NMR δ: −78.2 (CF_3_SO_3_). Accurate mass measurement: Calcd for C_28_H_19_I_2_O [M−CF_3_SO_3_]^+^: 624.9520. Found: 624.9515.

*(3-Iodo-1,4-diphenylnaphthalen-2-yl)(phenyl)iodonium triflate* (**5**). To a magnetically stirred suspension of DPIBF (**2**, 617 mg, 2.28 mmol) in anhydrous acetonitrile (5 mL), a solution of triflate **1** (575 mg, 1.14 mmol) in anhydrous acetonitrile (3 mL) was added dropwise under an Ar atmosphere and the mixture was vigorously stirred at room temperature for 20 h. The orange solution was concentrated in vacuo at 55 °C. In a certain moment the residue became dark brown. Crystallization of the above residue (1.31 g) from CH_2_Cl_2_/Et_2_O (3:10, 6.5 mL) gave a solid (1.02 g) mixture of triflate **5** and 1,2-phenylenebis(phenylmethanone) (**4**). After two crystallizations from CH_2_Cl_2_/toluene 3:10 (5.2 mL), triflate **5** (477 mg, 54% yield) was obtained as a beige solid, mp 207–208 °C (dec). IR (ATR) ν: 3058, 1490, 1470, 1443, 1282, 1266, 1222, 1153, 1023, 990, 835, 766, 722, 698, 666, 633, 599 cm^–1^. ^1^H-NMR (500 MHz) δ: 7.24–7.28 (complex signal, 4H, C1-Ar-H_ortho_ and C4-Ar-H_ortho_), 7.37–7.42 (complex signal, 2H, Ar-H_meta_ Ar-I^+^), 7.42–7.47 (complex signal, 4H, 5-H, 6-H, 7-H, 8-H), 7.51–7.62 (complex signal, 7H, C1-Ar-H_meta_, C4-Ar-H_meta_, C1-Ar-H_para_, C4-Ar-H_para_, and C2-I^+^-Ar-H_para_), 7.73 (dd, *J* = 8.5 Hz, *J′* =1.0 Hz, 2H, Ar-H_ortho_ ArI^+^). ^13^C-NMR (125.8 MHz) δ: 105.4 (C, C3), 116.3 (C, C2-I^+^-Ar-C_ipso_), 120.3 (C, q, *J* = 320 Hz, CF_3_SO_3_), 128.26 (CH), 128.30 (CH), 128.8 (CH), 129.3 (CH), 130.0 (CH) and 130.1 (CH) (C5, C6, C7, C8, C1-Ar-C_para_ and C4-Ar-C_para_), 128.9 (CH), 129.1 (CH), 129.2 (CH) and 129.9 (CH) (C1-Ar-C_ortho_, C4-Ar-C_ortho_, C1-Ar-C_meta_ and C4-Ar-C_meta_), 129.7 (C, C2), 131.9 (CH, Ar-C_meta_ Ar-I^+^), 132.4 (CH, C2-I^+^-Ar-C_para_), 132.5 (C) and 134.6 (C) (C4a and C8a), 134.4 (CH, C2-I^+^-Ar-C_ortho_), 141.7 (C, C1-Ar-C_ipso_), 145.2 (C, C4-Ar-C_ipso_), 149.3 (C) and 149.8 (C) (C1 and C4). ^19^F-NMR δ: −78.2 (CF_3_SO_3_). Accurate mass measurement: Calcd for C_28_H_19_I_2_ [M–CF_3_SO_3_]^+^: 608.9571. Found: 608.9567. Anal. Calcd for C_29_H_19_F_3_I_2_O_3_S·0.1CH_2_Cl_2_: C 45.58; H 2.52; I 33.10; F 7.43. Found: C 45.35; H 2.50; I 32.60; F 7.16.

X-Ray diffraction data for triflate **5**. A prismatic crystal (0.1 × 0.1 × 0.2 mm) was selected and mounted on a MAR345 diffractometer with an image plate detector. Unit-cell parameters were determined from 177 reflections (3 < θ < 31°) and refined by least-squares method. Intensities were collected with graphite monochromatized MoKα radiation; 23,699 reflections were measured in the range 1.91 ≤ θ ≤ 32.36. 8,297 of which were non-equivalent by symmetry [Rint (on I) = 0.058]. Reflections (5,839) were assumed as observed by applying the condition I >2σ(I). Lorentz-polarization but no absorption corrections were made. The structure was solved by direct methods, using SHELXS computer program and refined by full-matrix least-squares method with SHELX-97 computer program [8], using 23,699 reflections (very negative intensities were not assumed). The minimized function was Σw||Fo|^2^ – |Fc|^2^|^2^, where w = [σ^2^(I) + (0.0339P)^2^ + 1.5431P]^−1^, and P = (|Fo|^2^+ 2|Fc|^2^)/3, f, f', and f" were taken from International Tables of X-ray Crystallography [9]. All H atoms were computed and refined, using a riding model, with an isotropic temperature factor equal to 1.2 times the equivalent temperature factor of the atom to which it is linked. The final R (on F) factor was 0.050, wR(on |F|^2^) = 0.108 and goodness of fit = 1.138 for all observed reflections. Number of refined parameters was 343. Max. shift/esd = 0.00, mean shift/esd = 0.00. Max. and min. peaks in final difference synthesis were 0.970 and −0.582 eÅ^−3^, respectively [10].

*2,3-Diiodo-1,4-diphenyl-1,4-dihydro-1,4-epoxynaphthalene* (**6**). To a cold (−35 to −40 °C), magnetically stirred solution of NaI (23 mg, 0.154 mmol) and CuI (29 mg, 0.154 mmol) in anhydrous acetonitrile (2.3 mL) under an Ar atmosphere, triflate **3** (120 mg, 0.154 mmol) was added. The mixture was allowed to warm to room temperature and was stirred overnight at this temperature. The solvent and volatile products were eliminated under reduced pressure and the residue was extracted with CH_2_Cl_2_ (4 × 2 mL). The combined organic extracts were filtered through a PTFE filter and were concentrated under reduced pressure to give diodide **6** as a light yellow solid (74 mg, 87% yield). The analytical sample of **6** was obtained as a light yellow solid (58 mg, 69% yield of crystallized product) by crystallization from CH_2_Cl_2_/MeOH 5:2 (0.7 mL), m.p. 158–160 °C (dec.). IR ν 3053, 1599, 1544, 1492, 1449, 1342, 1327, 1304, 1132, 1025, 998, 943, 917, 905, 854, 761, 755, 738, 705, 698, 674, 652, 590 cm^−1^. ^1^H-NMR δ 7.10–7.12 [m, 4H, 6(7)-H], 7.46–7.50 [complex signal, 4H, 5(8)-H and phenyl-H*_para_*], 7.52–7.56 (m, 4H, phenyl-H*_meta_*), 7.89–7.92 (dm, *J*= 8.0 Hz, 4H, phenyl-H*_ortho_*). ^13^C-NMR δ 96.7 [(C, C1(4)], 121.2 [CH, C5(8)], 125.7 [CH, C6(7)], 126.4 [(C, C2(3)], 128.0 (CH, phenyl-C*_ortho_*), 128.2 (CH, phenyl-C*_meta_*), 128.9 (CH, phenyl-C*_para_*), 134.1 (C, phenyl-C*_ipso_*), 148.3 [C, C4a(8a)]. Accurate mass measurement: Calcd for C_22_H_15_I_2_O [M+H]^+^: 548.9207. Found: 548.9188; Calcd for [M+H−I]^+^: 422.0162. Found: 422.0158. Anal. Calcd for C_22_H_14_I_2_O: C 48.20, H 2.57, I 46.30. Found: C 48.25; H 2.64; I 46.22.

X-ray diffraction data for diiodide **6**. A prismatic crystal (0.1 × 0.1 × 0.2 mm) was selected and mounted on a MAR345 diffractometer with an image plate detector. Unit-cell parameters were determined from 289 reflections (3 < θ < 31°) and refined by least-squares method. Intensities were collected with graphite monochromatized MoKα radiation; 10,713 reflections were measured in the range 1.92 ° θ ° 32.03. 4,303 of which were non-equivalent by symmetry (Rint (on I) = 0.045). 3,705 reflections were assumed as observed by applying the condition I >2σ(I). Lorentz-polarization but no absorption corrections were made. The structure was solved by direct methods, using SHELXS computer program and refined by full-matrix least-squares method with SHELX-97 computer program [8], using 10,713 reflections (very negative intensities were not assumed). The minimized function was Σw||Fo|^2^–|Fc|^2^|^2^, where w = [σ^2^(I) + (0.0258P)^2^ + 1.0315P]^–1^, and P = (|Fo|^2^ + 2|Fc|^2^)/3, f, f', and f" were taken from International Tables of X-ray Crystallography [9]. All H atoms were computed and refined, using a riding model, with an isotropic temperature factor equal to 1.2 times the equivalent temperature factor of the atom to which it is linked. The final R (on F) factor was 0.031, wR(on |F|^2^) = 0.072 and goodness of fit = 1.171 for all observed reflections. Number of refined parameters was 226. Max. shift/esd = 0.00, mean shift/esd = 0.00. Max. and min. peaks in final difference synthesis were 0.395 and −0.620 eÅ^−3^, respectively [10].

*2,3-Diiodo-1,4-diphenylnaphthalene* (**7**). To a cold (−35 to −40 °C), magnetically stirred solution of NaI (92 mg, 0.61 mmol) and CuI (117 mg, 0.61 mmol) in anhydrous acetonitrile (10 mL) under an Ar atmosphere, triflate **5** (455 mg, 0.60 mmol) was added. The mixture was allowed to warm to room temperature and was stirred overnight at this temperature. The solution was separated from the inorganic salts by decantation. The salts were washed with CH_2_Cl_2_ (2 × 2 mL). The combined organic phases were concentrated under reduced pressure to give a solid residue (758 mg) that was subjected to column chromatography (silica gel, 30 g, hexane/EtOAc mixtures) to give the volatile iodobenzene (12 mg, 10%) on elution with hexane and product **7** as a white solid, m.p. 269–271 °C (328 mg, quantitative yield) on elution with hexane/EtOAc 99:1. IR ν 3058, 2921, 2844, 1490, 1480, 1439, 1364, 1270, 1260, 1107, 1073, 1027, 840, 764, 719, 697, 667, 599 cm^−1^. ^1^H-NMR δ 7.23–7.27 (dm, *J* = 6.8 Hz, 4H, Ar-H*_ortho_* phenyl), 7.28–7.32 [m, 2H, 6(7)-H], 7.30–7.35 [m, 2H, 5(8)-H], 7.48 (tm, *J* = 8.5 Hz, 2H, Ar-H*_para_* phenyl), 7.55 (m, 4H, Ar-H*_meta_* phenyl). ^13^C-NMR δ 112.7 [C, C2(3)], 126.9 [CH, C6(7)], 127.8 [CH, C5(8)], 128.0 (CH, Ar-C*_para_* phenyl), 128.6 (CH, Ar-C*_meta_* phenyl), 129.6 (CH, Ar-C*_ortho_* phenyl), 132.4 [C, C4a(8a)], 146.4 (C, Ar-C*_ipso_* phenyl), 146.7 [C, C1(4)]. Anal. Calcd for C_22_H_14_I_2_: C 49.65, H 2.65, I 47.69. Found: C 49.81; H 2.71; I 47.55.

## 4. Conclusions

Triflate **1** reacts with DPIBF at room temperature to give the corresponding Diels-Alder adduct **3** in high yield. Adduct **3** is thermally unstable, decomposing in solution at about 55 °C. Reaction of triflate **1** with an excess (2 equiv.) of DPIBF in acetonitrile solution at 55 °C gave a mixture of dione **4** and triflate **5**. A similar oxygen transfer among triflate **3** and dimethylsulfide was not observed. To the best of our knowledge, no similar oxygen transfer has been ever described. Also, the easy access to diiodide **7** as compared with the described procedure [7] might be of interest in connection with the preparation of rubrenes [6,11].

## Supplementary Material

IR and NMR spectra of triflates **3** and **5** and diiodides **6** and **7**. Detailed information can be accessed at: http://www.mdpi.com/1420-3049/17/8/8795/s1.
