# Supplementary material for: On the Reaction of 1,3-Diphenylisobenzofuran and (2-Iodoethynyl)(phenyl)iodonium Triflate. A Unique Case of Oxygen Transfer from the Diels-Alder Adduct to the Diene †
Source: Molecules. 2012 Jul 25;17(8):8795–803. doi: 10.3390/molecules17088795 (PMC6268445; doi:10.3390/molecules17088795)
Supplement: Supplementary file 1 [file molecules-17-08795-s001.pdf]

On the reaction of 1,3-diphenylisobenzofuran and (2-iodoethynyl)(phenyl)iodonium triflate. A unique case of oxygen transfer from Diels-Alder adduct to the diene.

Pelayo Camps,*[a] Tània Gómez,[a] David Lozano,[a] Teresa Calvet,[b] Mercè Font-Bardia[b][c]

[a]Laboratori de Química Farmacèutica (Unitat Associada al CSIC), Facultat de Farmàcia, and Institut de Biomedicina de la Universitat de Barcelona (IBUB), Universitat de Barcelona, Av. Joan XXIII s/n, E-08028, Barcelona, Spain;

[b]Cristalografia, Mineralogia i Dipòsits Minerals, Universitat de Barcelona, Martí Franquès s/n. E-08028 Barcelona, Spain;

[c]Unitat de Difracció de RX, Centre Científic i Tecnològic de la Universitat de Barcelona (CCiTUB), Universitat de Barcelona, Solé i Sabarís 1-3. E-08028 Barcelona, Spain

E.mail: *camps@ub.edu*

Supporting information

Content

NMR and IR spectra of triflate **3**…………………………………………...……………...S2

NMR and IR spectra of triflate **5**…………………………………………….....………...S8

NMR and IR spectra of diiodide **6**………………………..……………………….…...S14

NMR and IR spectra of diiodide **7**……………………………………………….........S18

NMR and IR spectra of triflate 3

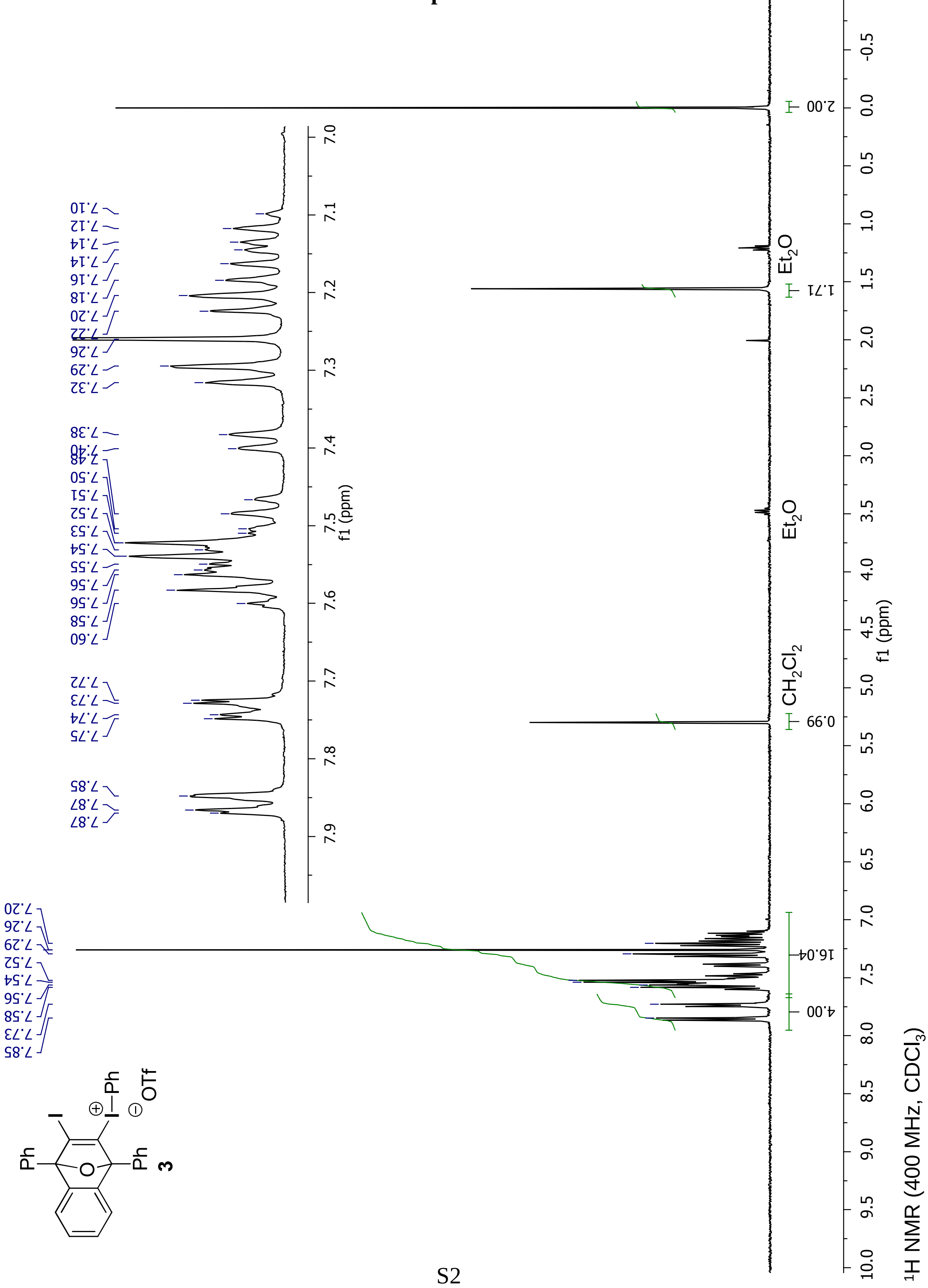

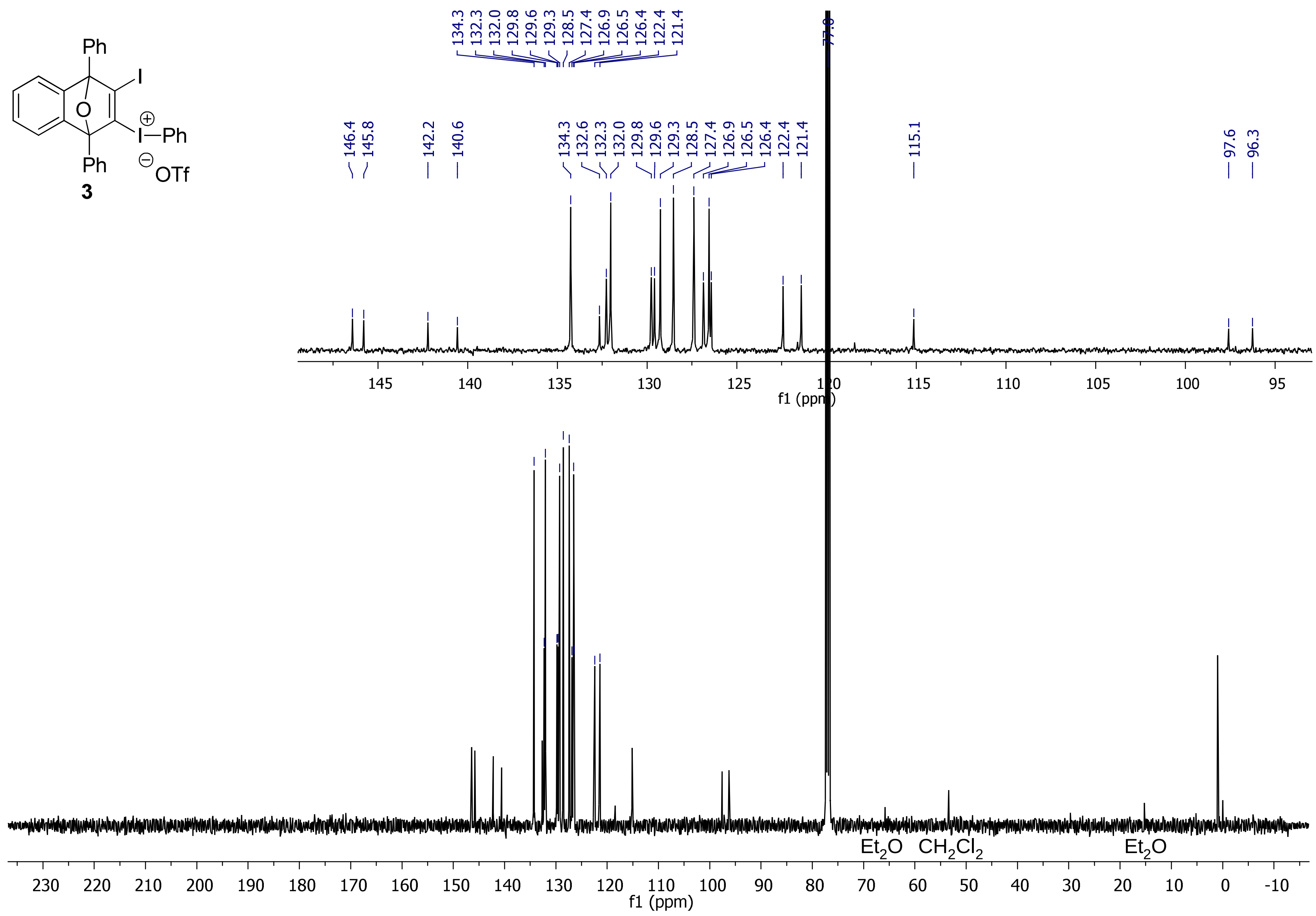

3

^{13}C NMR (100.6 MHz, $CDCl_3$)

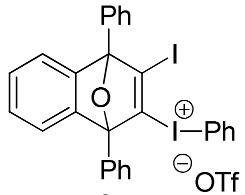

3

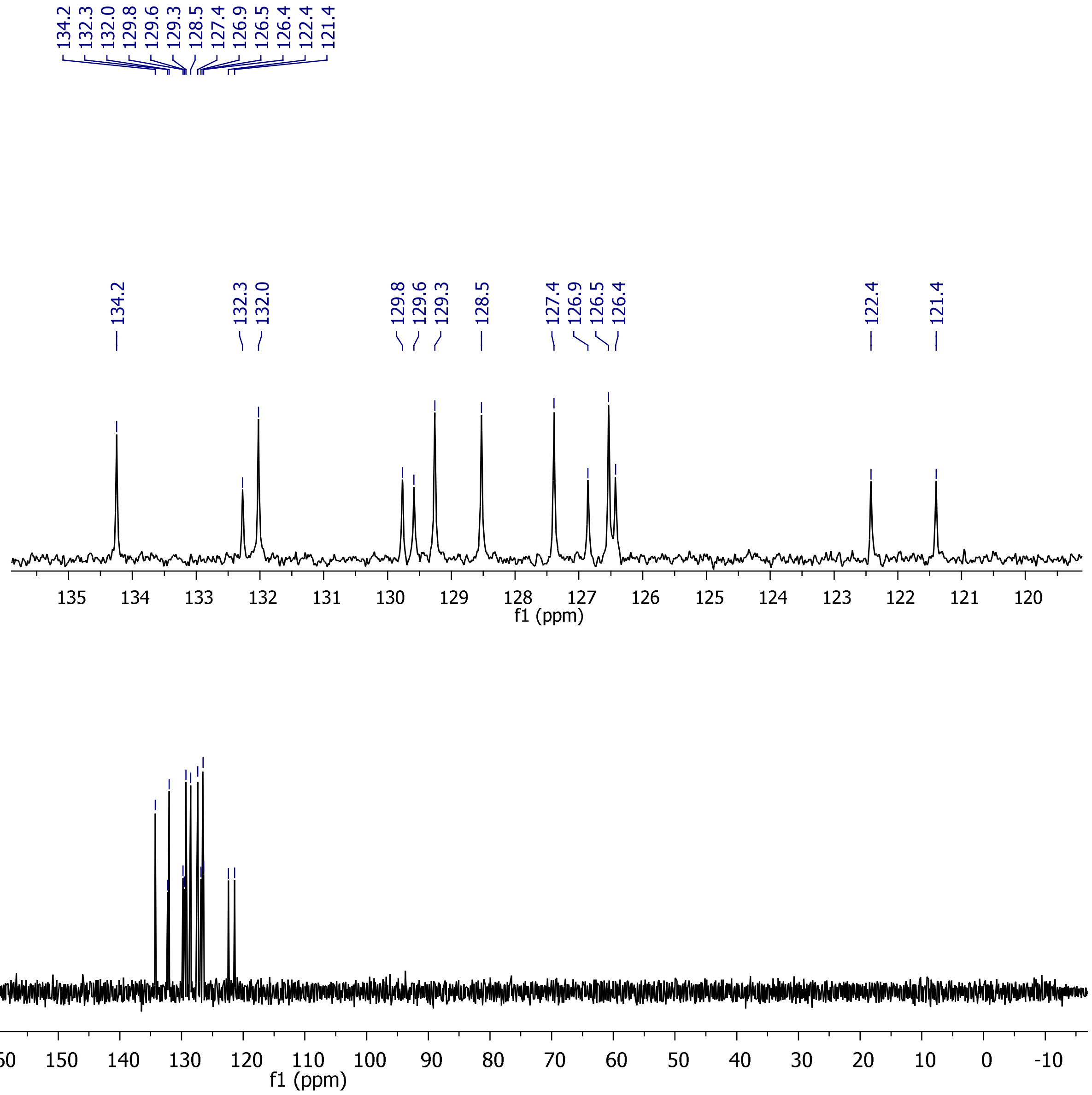

DEPT (100.6 MHz, $CDCl_3$)

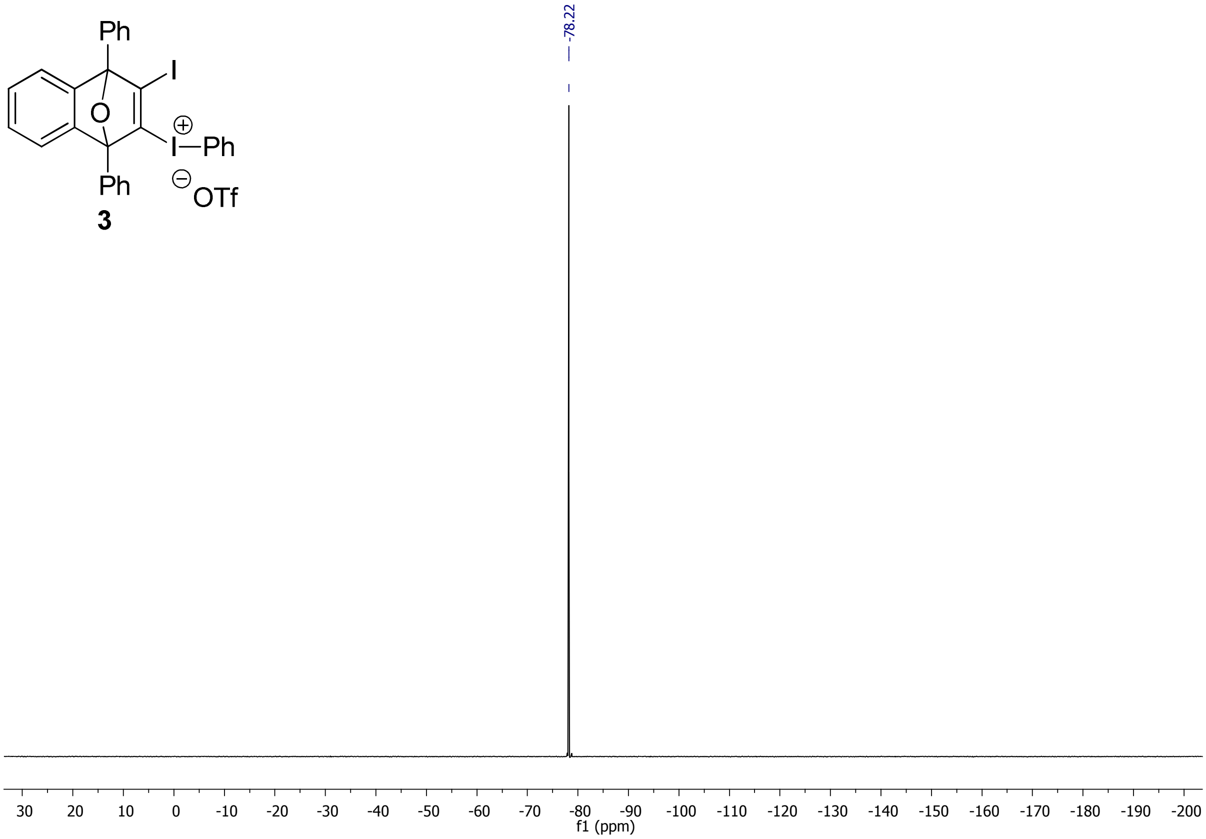

3

^{19}F NMR (376.29 MHz, $CDCl_3$)

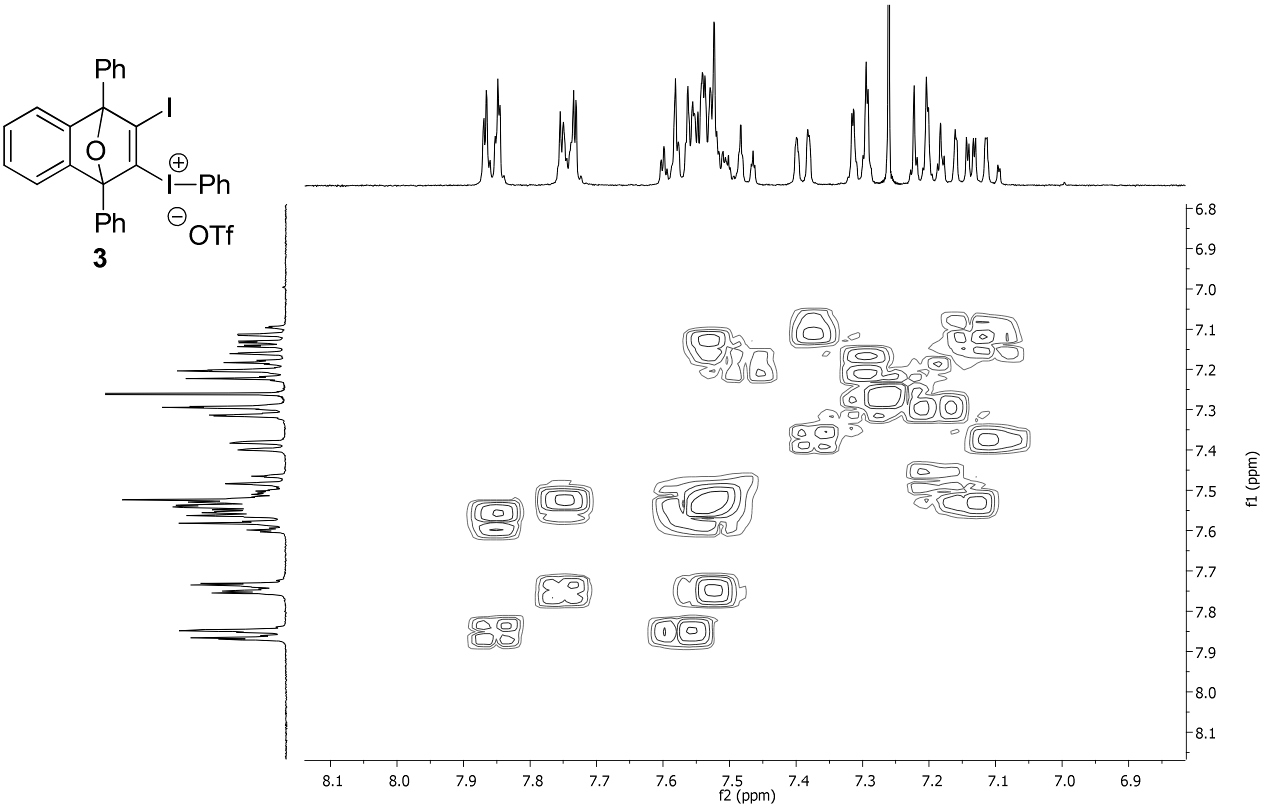

3

^{1}H/^{1}H COSY (400 MHz, $CDCl_3$)

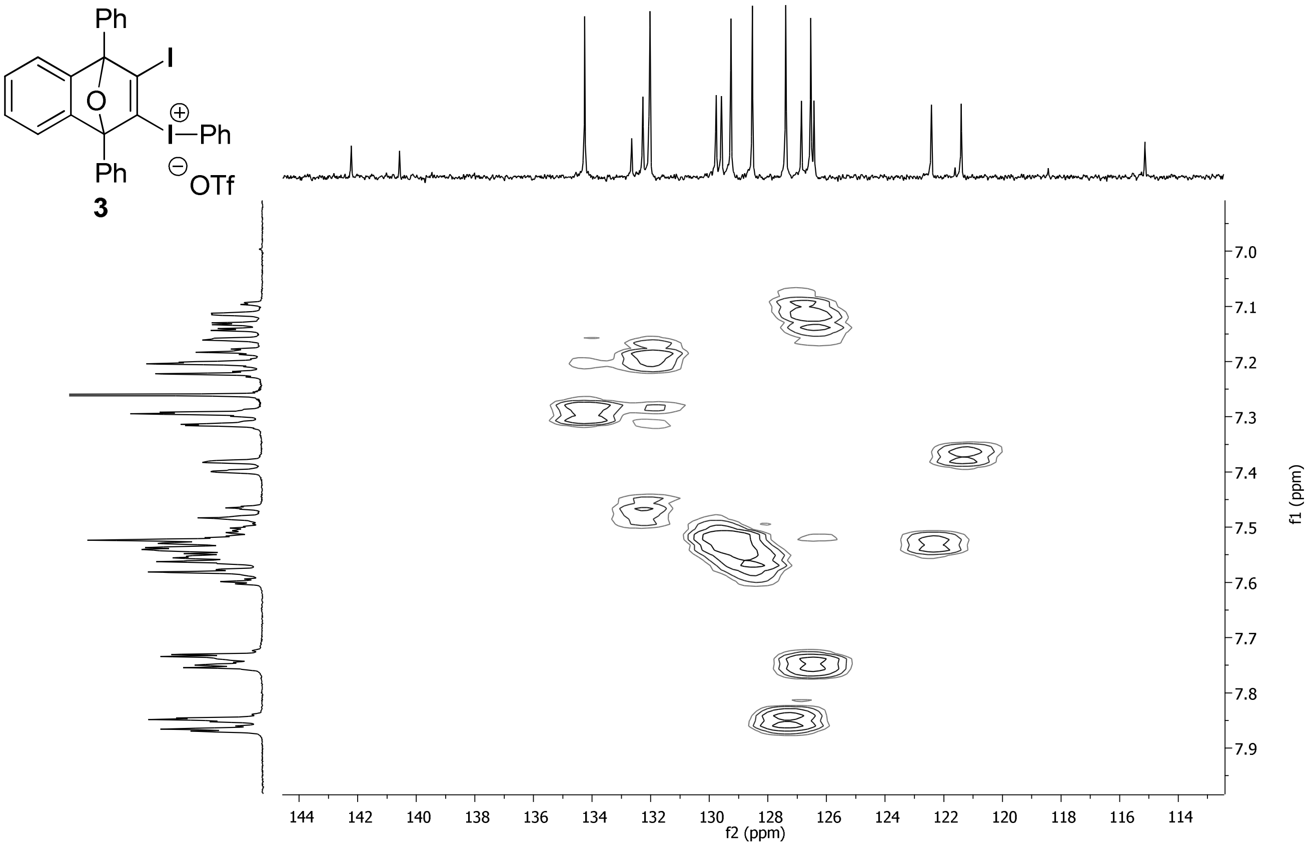

$^{1}H/^{13}C$ HETCOR (gHSQC sequence) (400 MHz, $CDCl_3$)

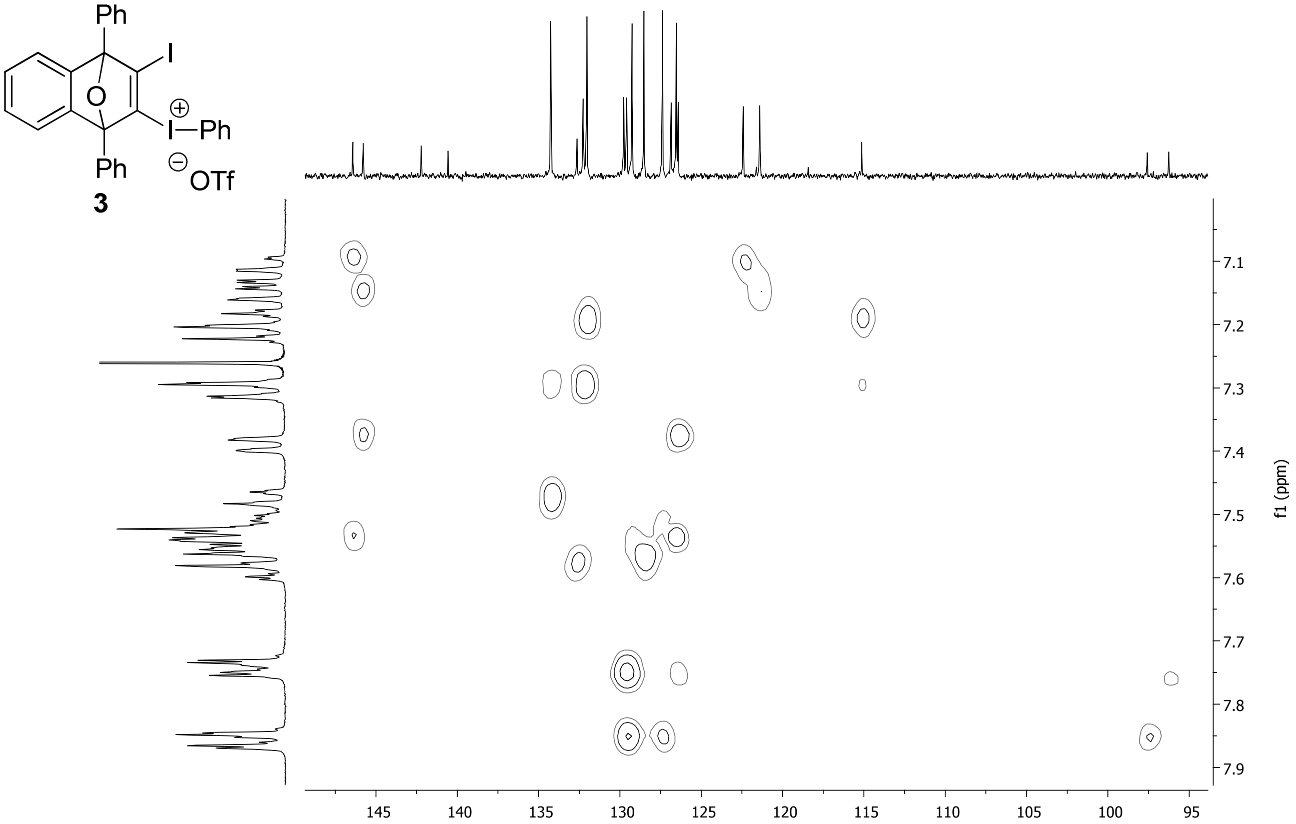

$^{1}H/^{13}C$ HETCOR (gHMBC sequence) (400 MHz, $CDCl_3$)

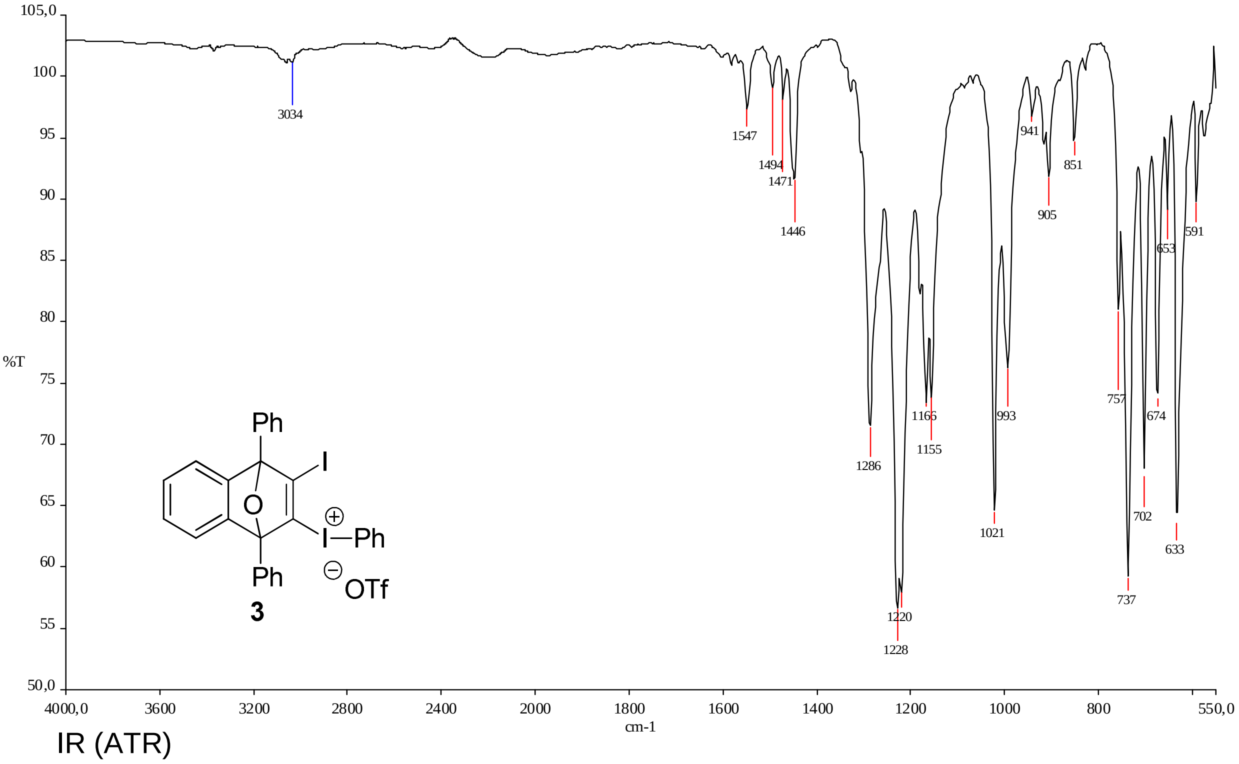
105,0
100
95
90
85
80
%T
75
70
65
60
55
50,0
4000,0
3600
3200
2800
2400
2000
1800
1600
1400
1200
1000
800
550,0
cm-1
3034
1547
1494
1471
1446
1286
1220
1228
1166
1155
1021
993
941
905
851
757
737
702
674
653
633
591
Ph
I
O
I—Ph
Ph
OTf
3

IR (ATR)

NMR and IR spectra of triflate 5

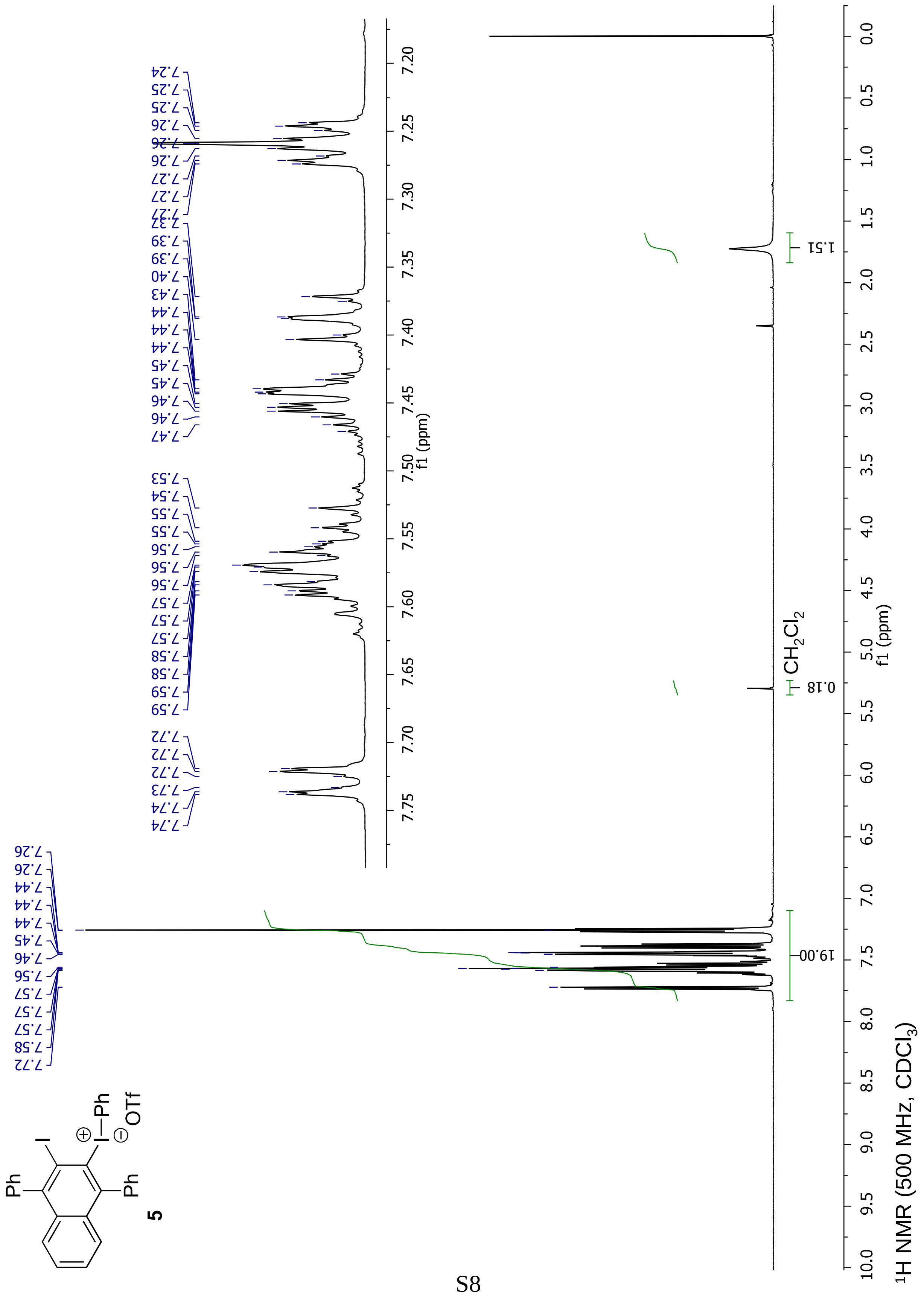

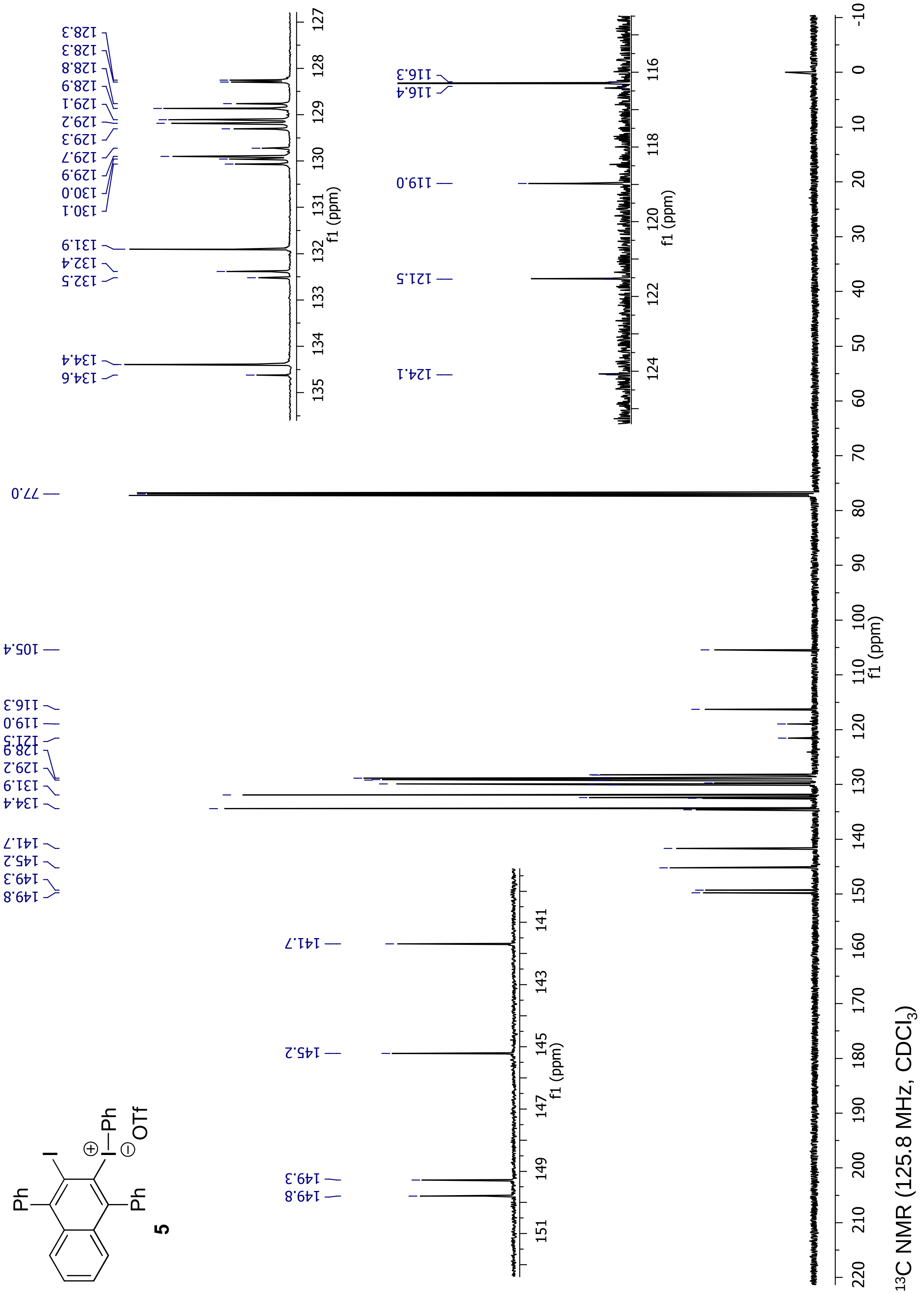

^{13}C NMR (125.8 MHz, $CDCl_3$)
5
f1 (ppm)

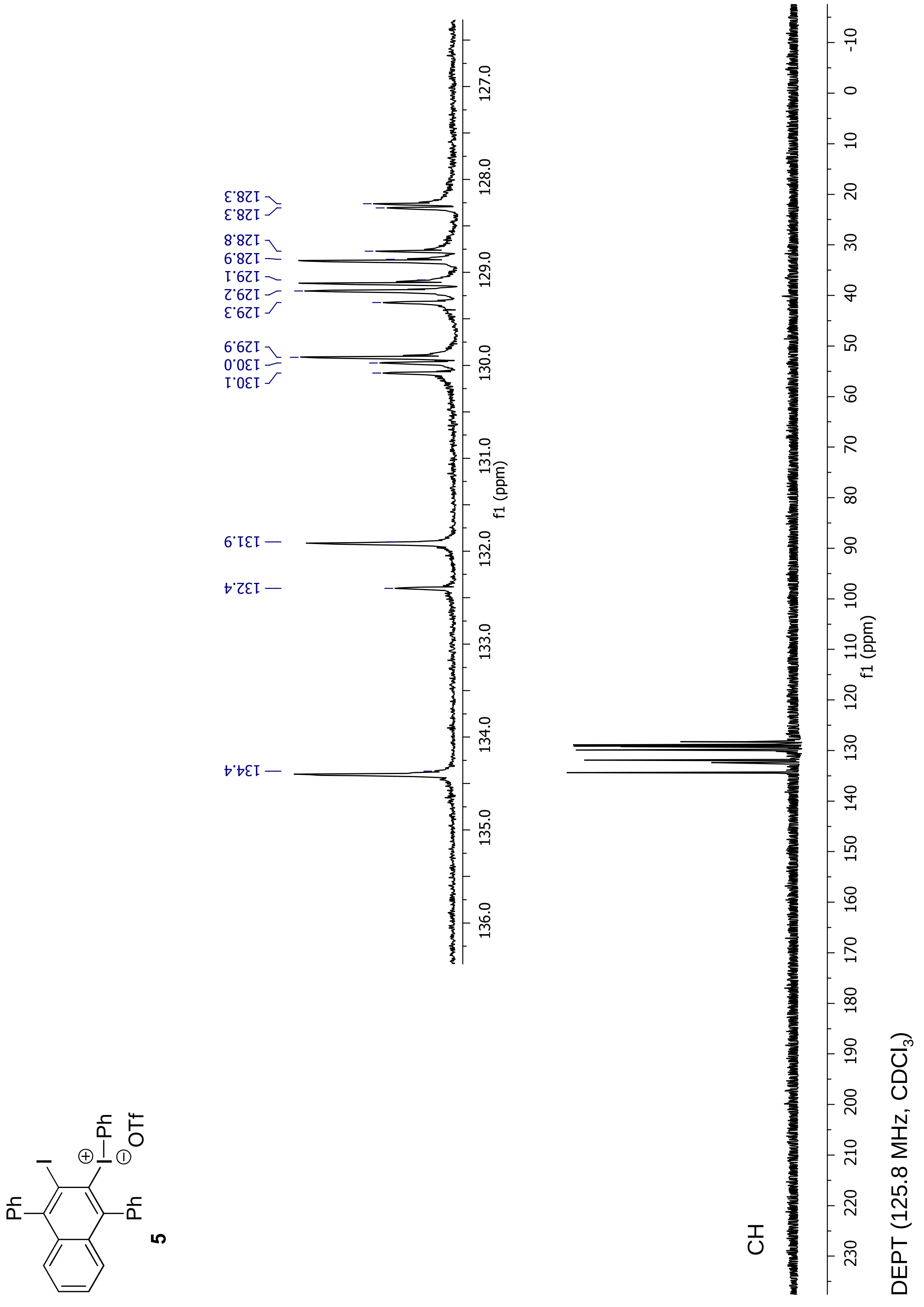
5
CH
DEPT (125.8 MHz, CDCl3)
f1 (ppm)
134.4
132.4
131.9
130.1
130.0
129.9
129.3
129.2
129.1
128.9
128.8
128.3
128.3

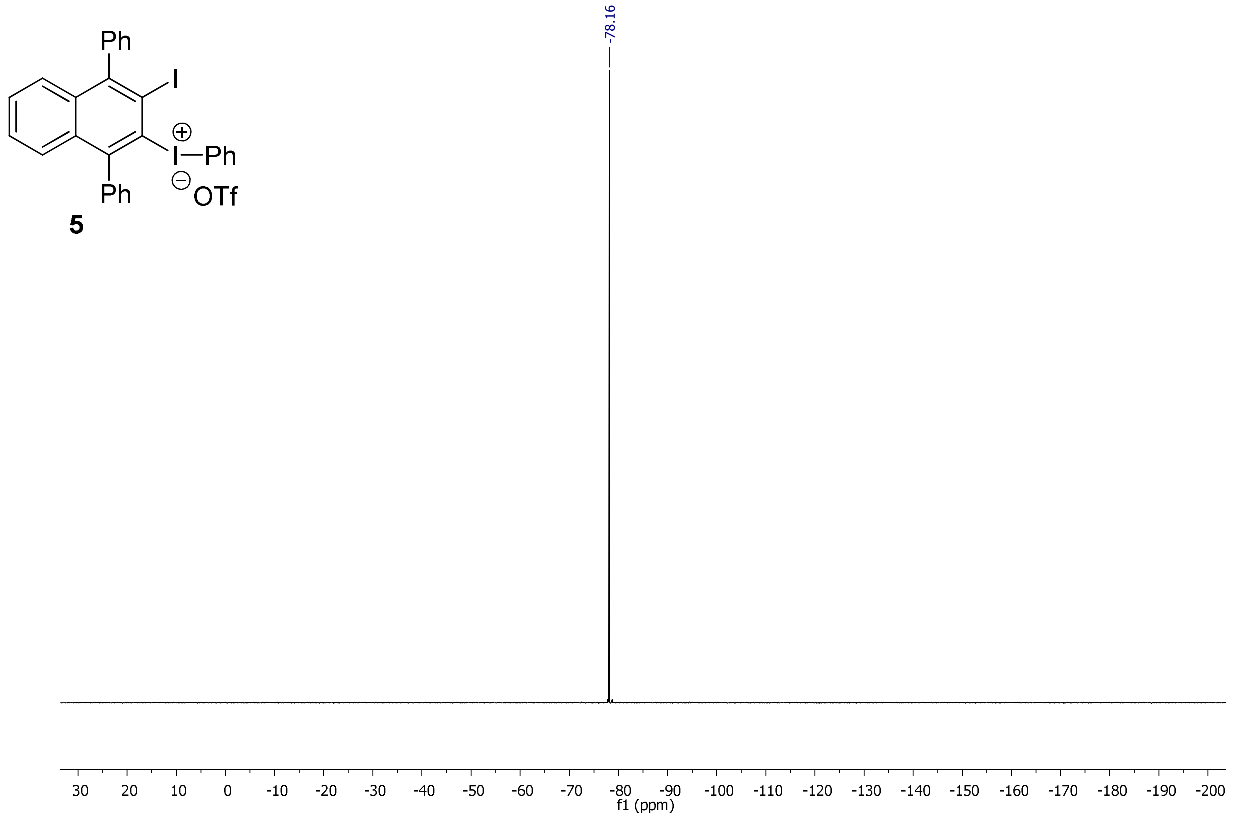

5

^{19}F NMR (376.29 MHz, $CDCl_3$)

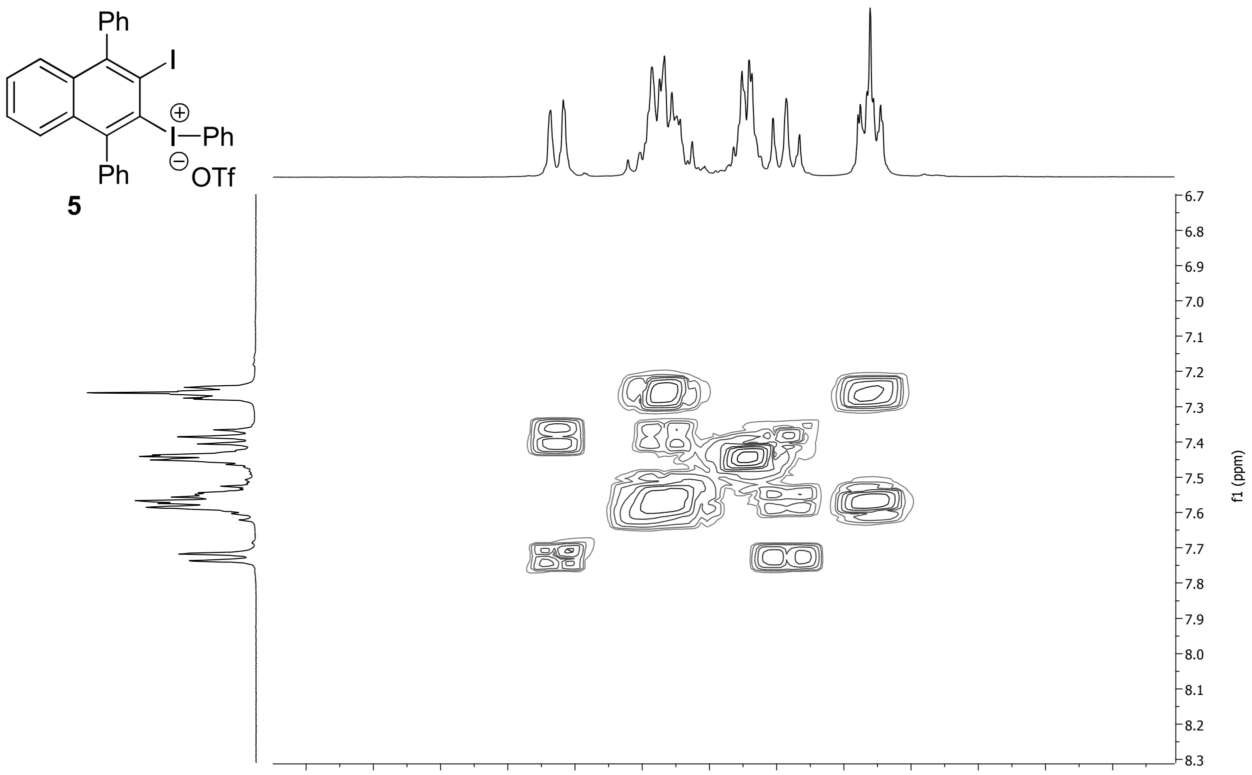

5

^{1}H/^{1}H COSY (400 MHz, $CDCl_3$)

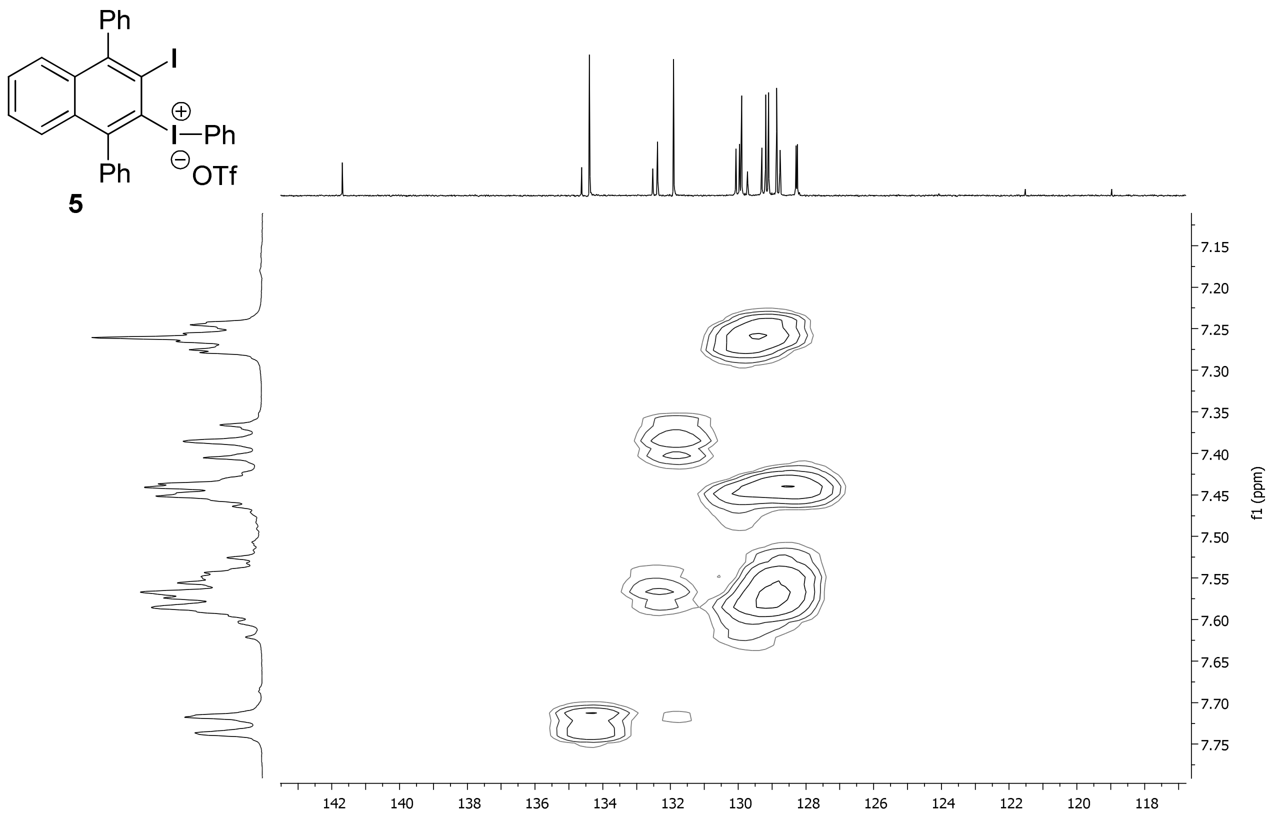

$^1H/^{13}C$ HETCOR (gHSQC sequence) (400 MHz, $CDCl_3$)

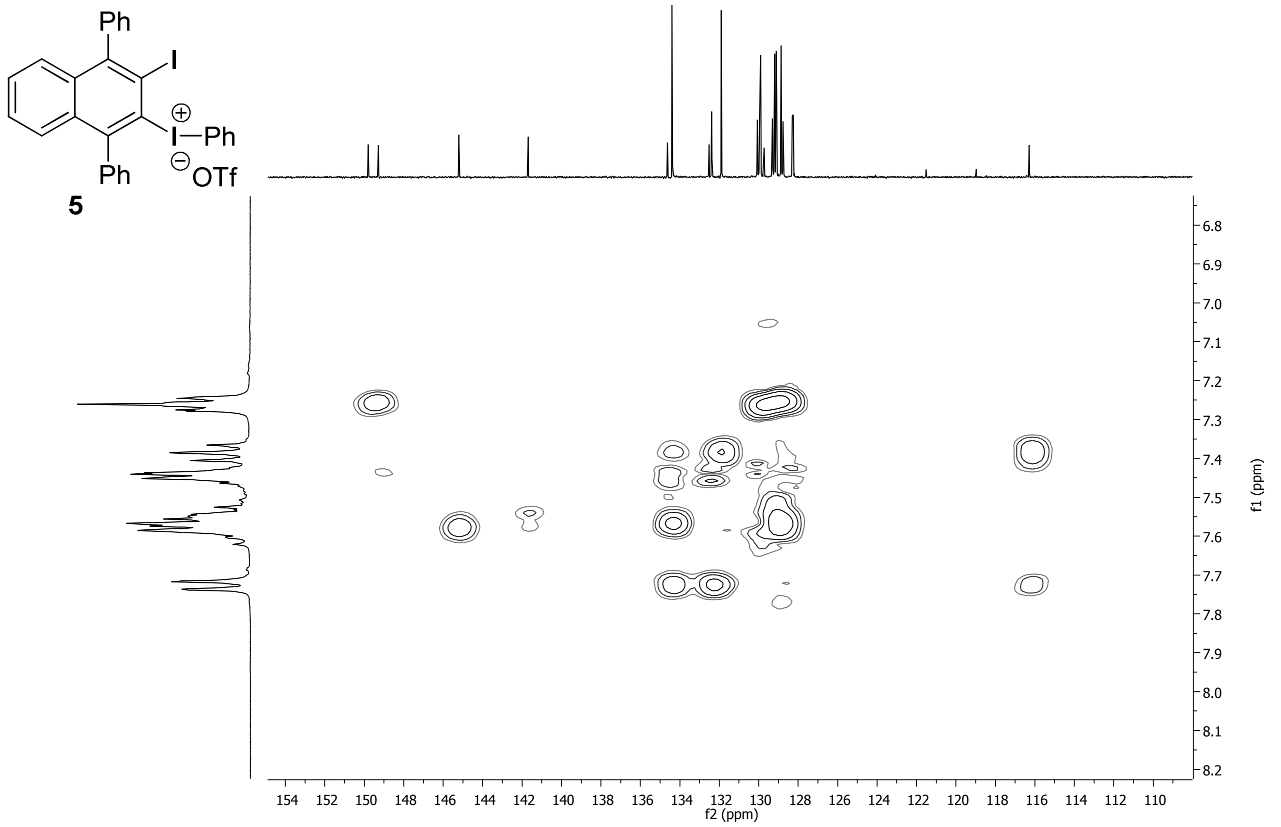

$^1H/^{13}C$ HETCOR (gHMBC sequence) (400 MHz, $CDCl_3$)

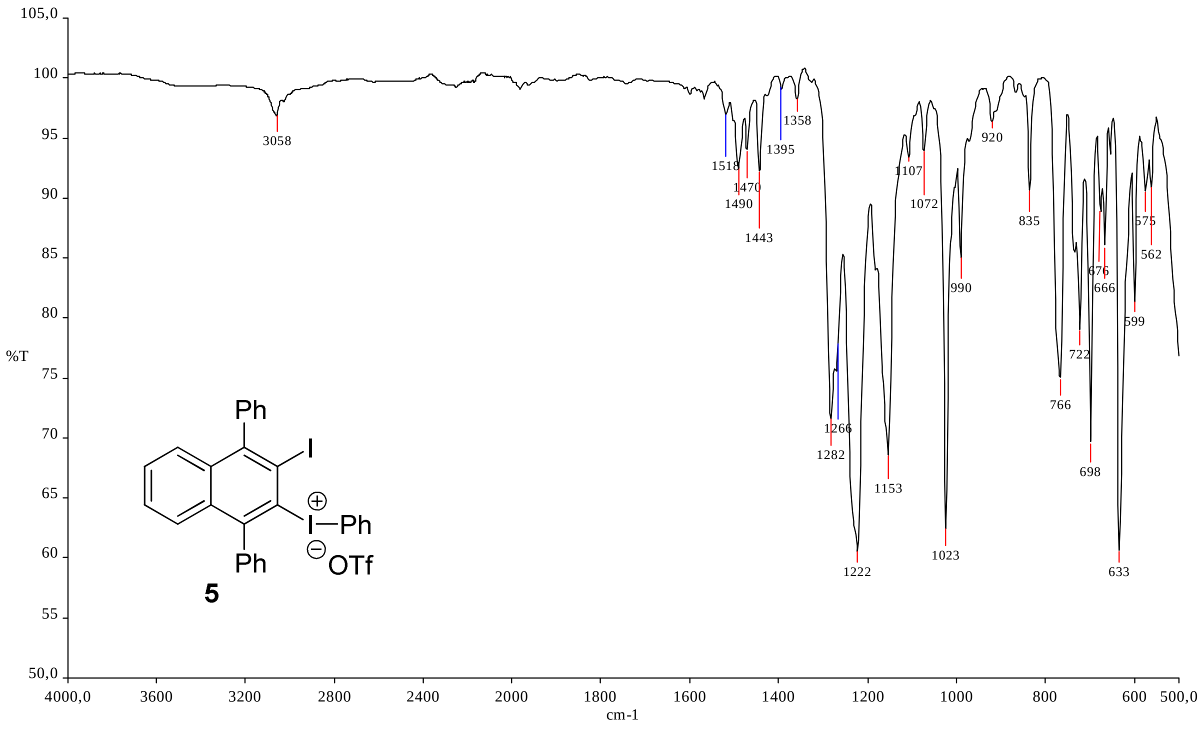
105,0
100
95
90
85
80
75
70
65
60
55
50,0
%T
4000,0
3600
3200
2800
2400
2000
1800
1600
1400
1200
1000
800
600
500,0
cm-1
3058
1518
1490
1470
1443
1395
1358
1282
1266
1222
1153
1107
1072
1023
990
920
835
766
722
698
676
666
633
599
575
562
Ph
I
I—Ph
OTf
5

IR (ATR)

NMR and IR spectra of diiodide 6

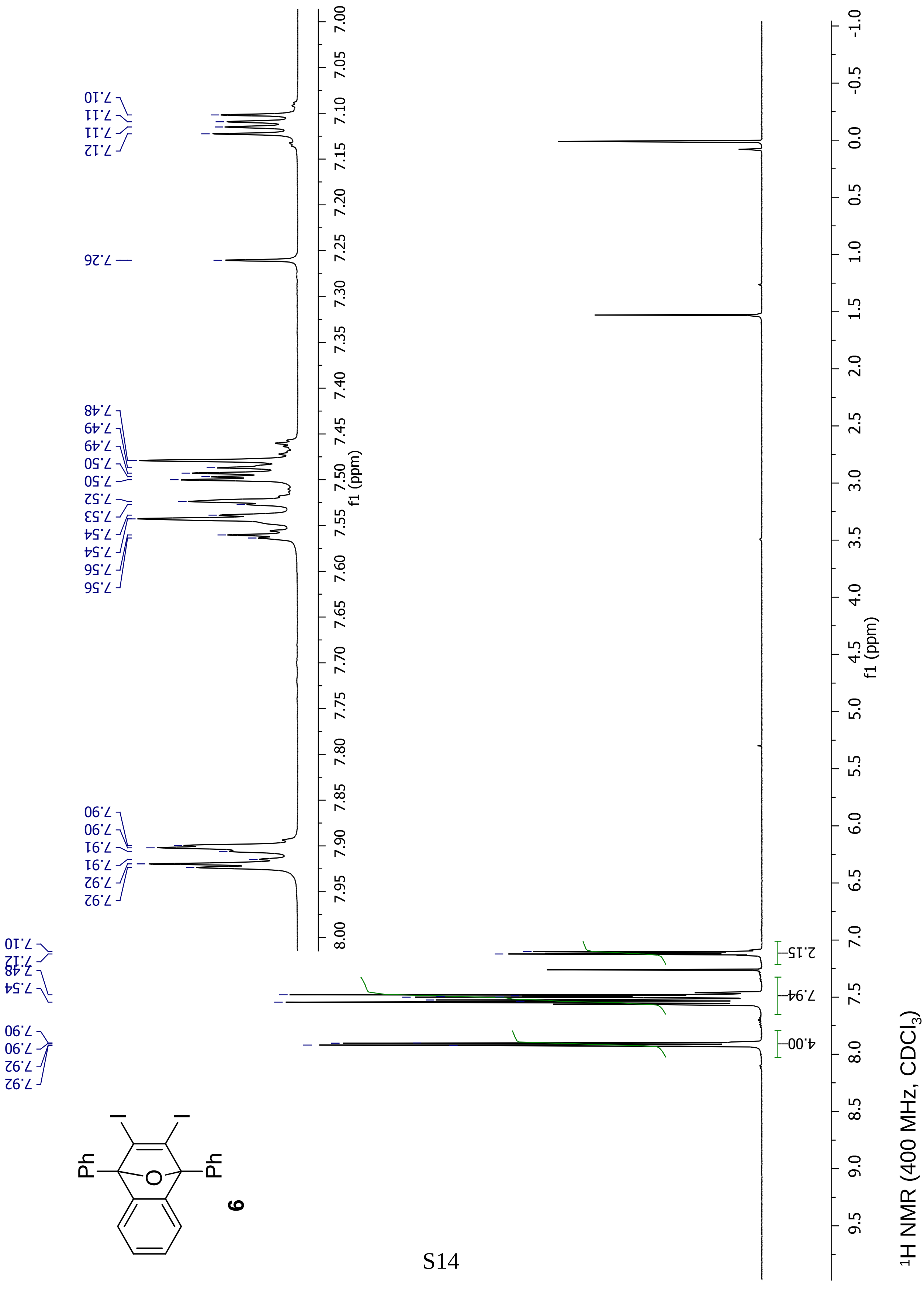

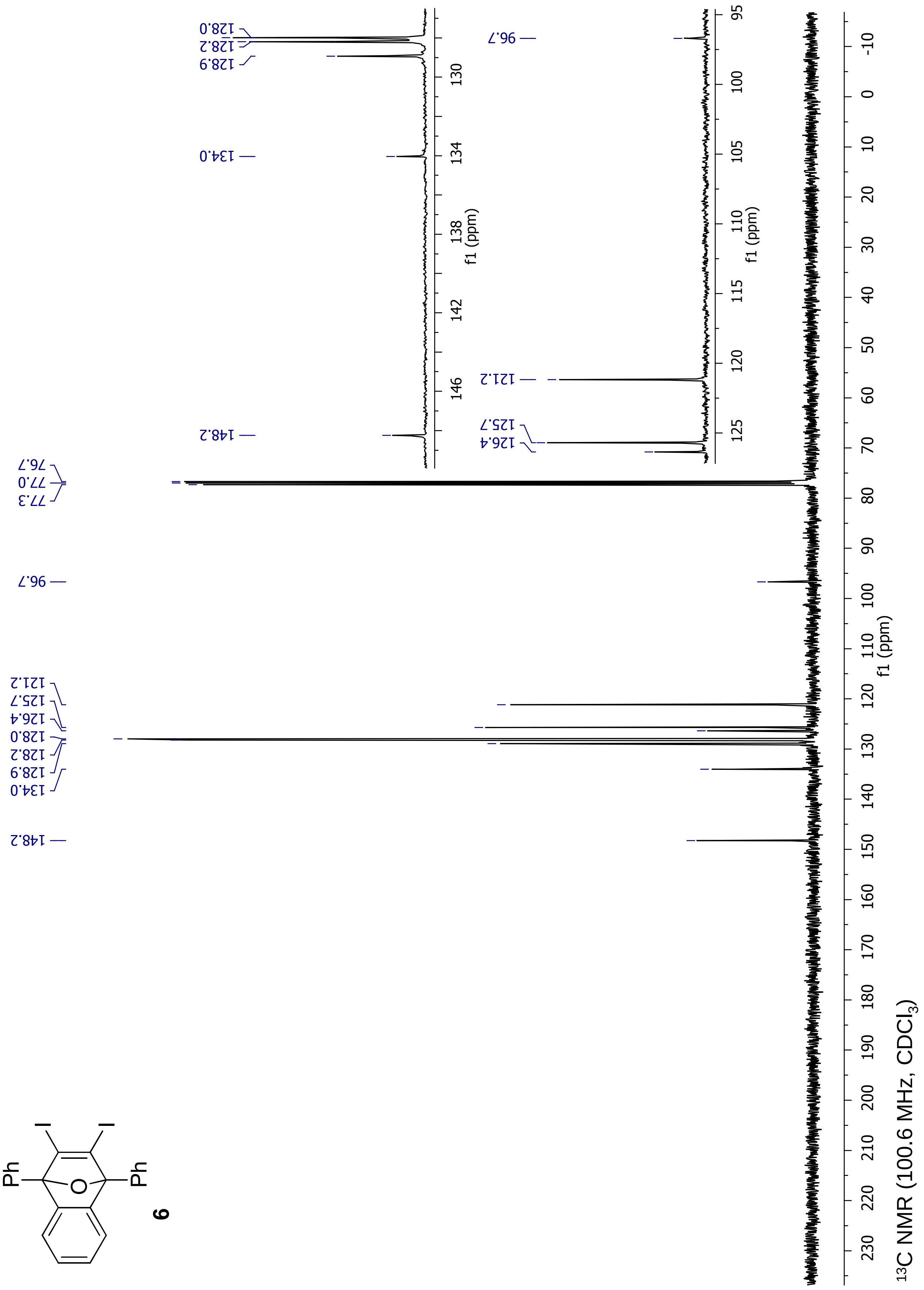
Ph
Ph
I
I
O
6
148.2
134.0
128.9
128.2
128.0
126.4
125.7
121.2
96.7
77.3
77.0
76.7
f1 (ppm)
13C NMR (100.6 MHz, CDCl3)

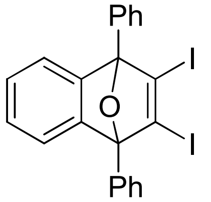

6

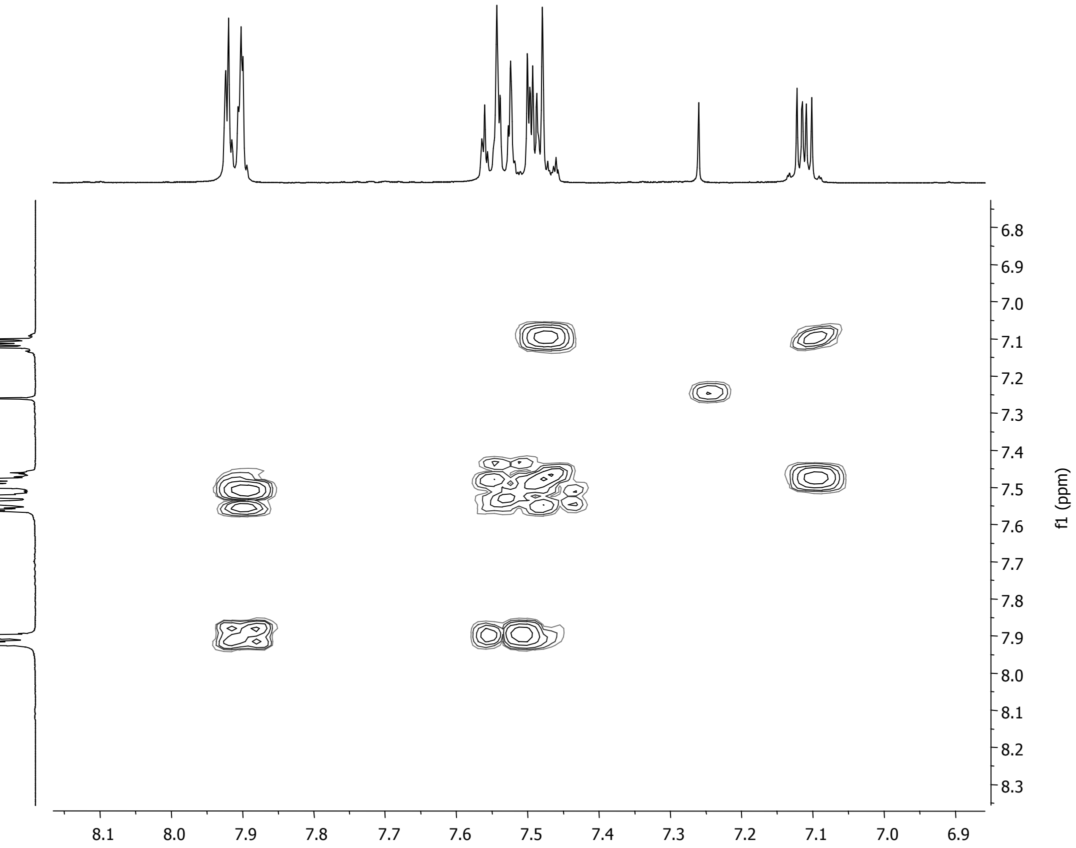

$^{1}H/^{1}H$ COSY (400 MHz, $CDCl_3$)

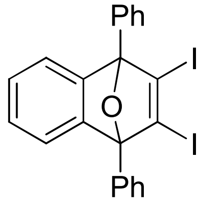

6

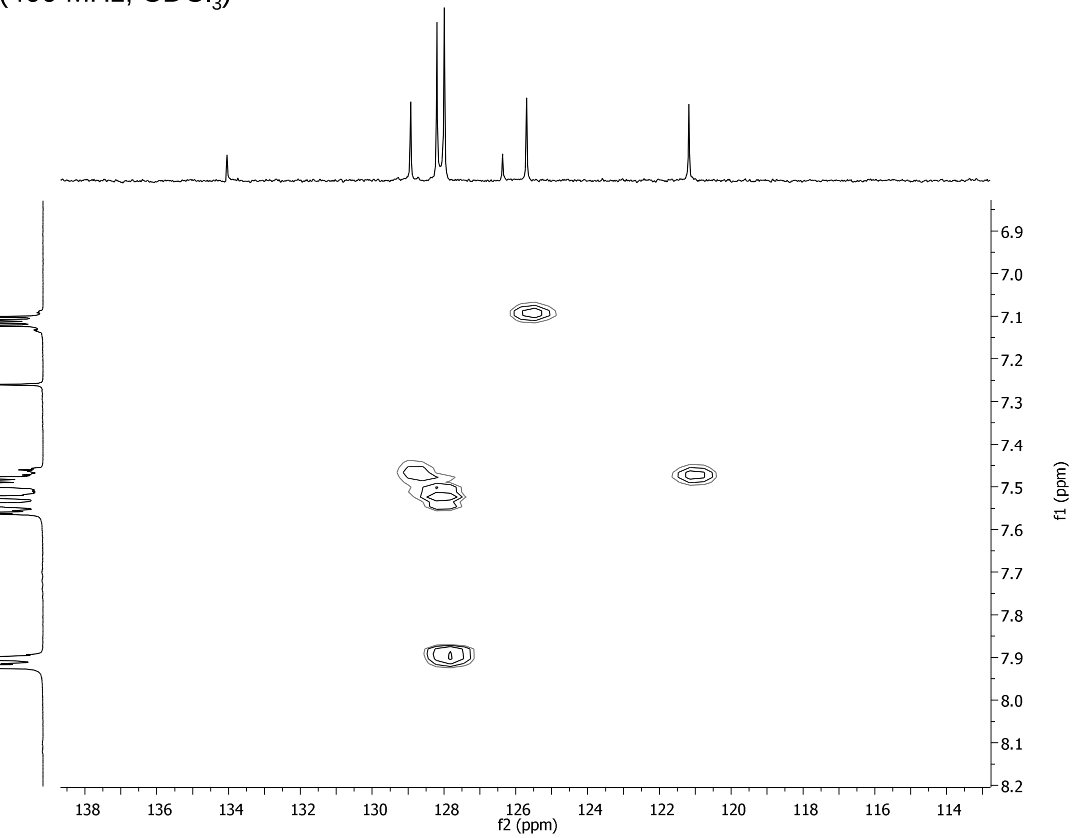

$^{1}H/^{13}C$ HETCOR (gHSQC sequence) (400 MHz, $CDCl_3$)

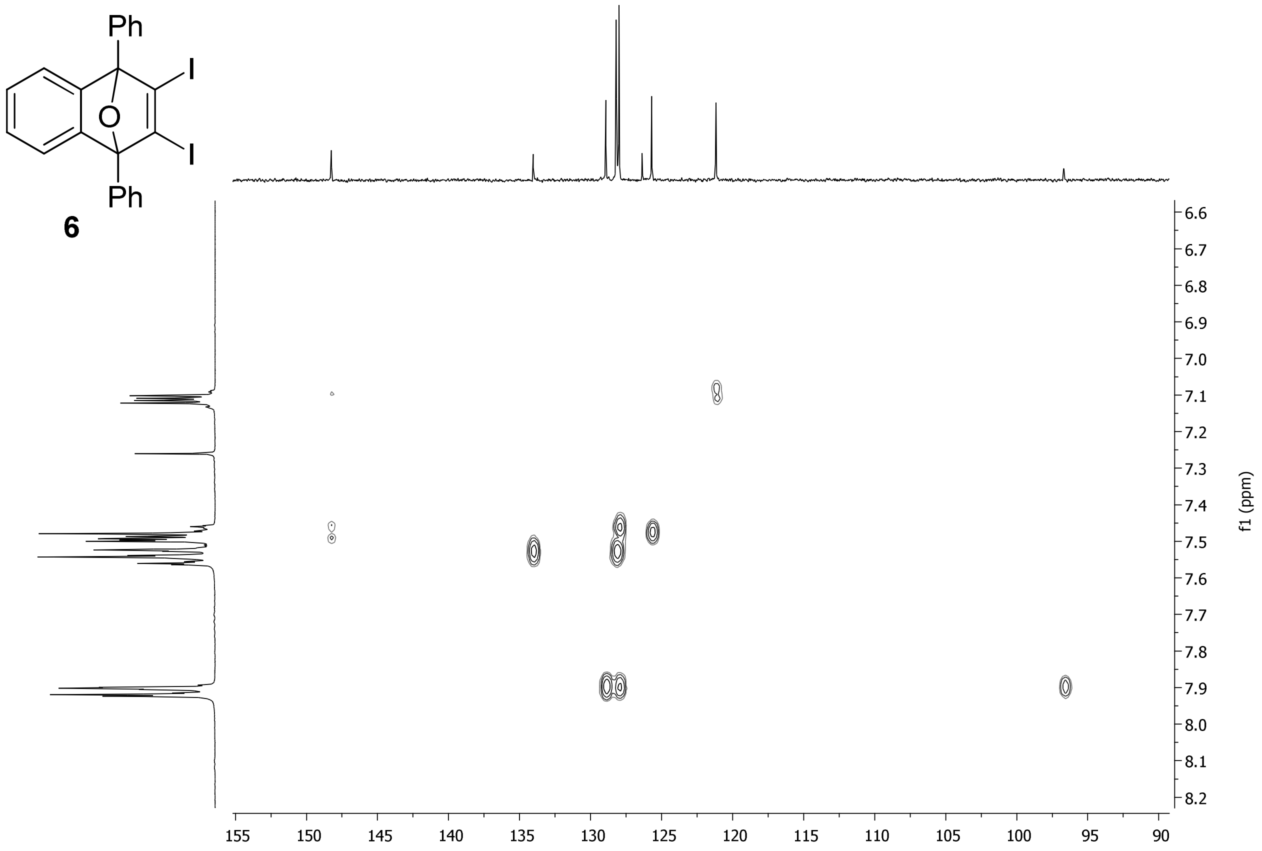

$^1H/^{13}C$ HETCOR (gHMBC sequence) (400 MHz, $CDCl_3$)

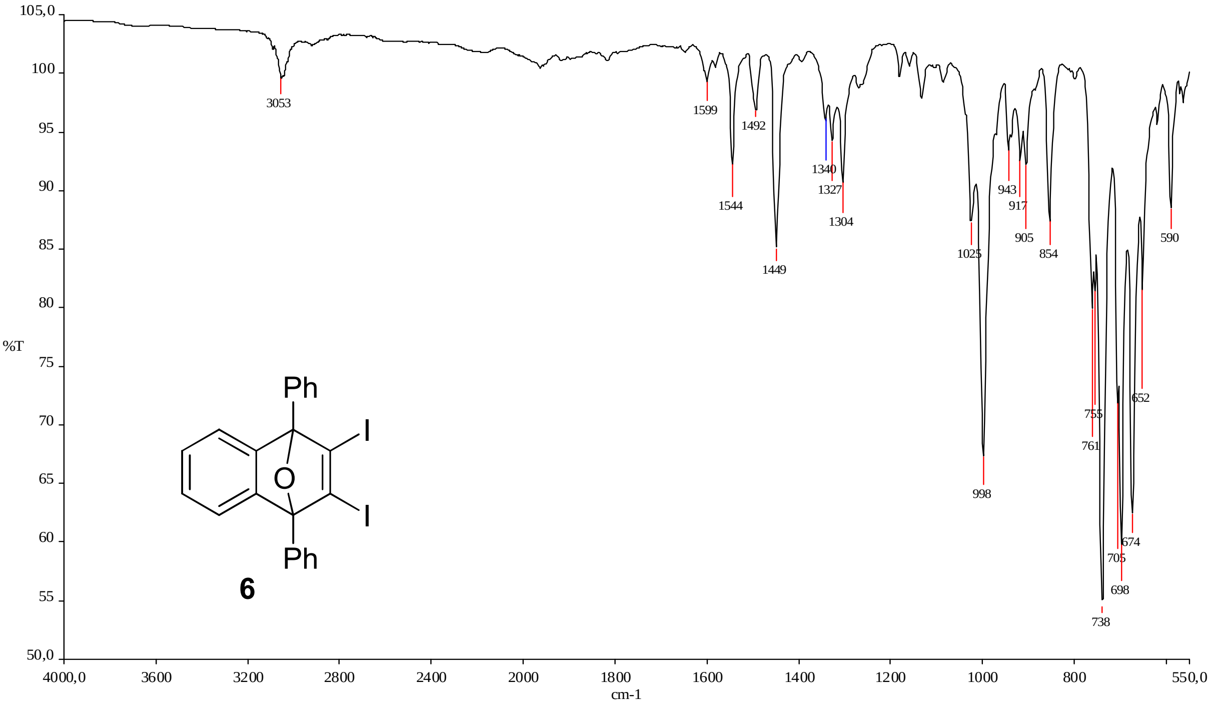

IR (ATR)

NMR and IR spectra of diiodide 7

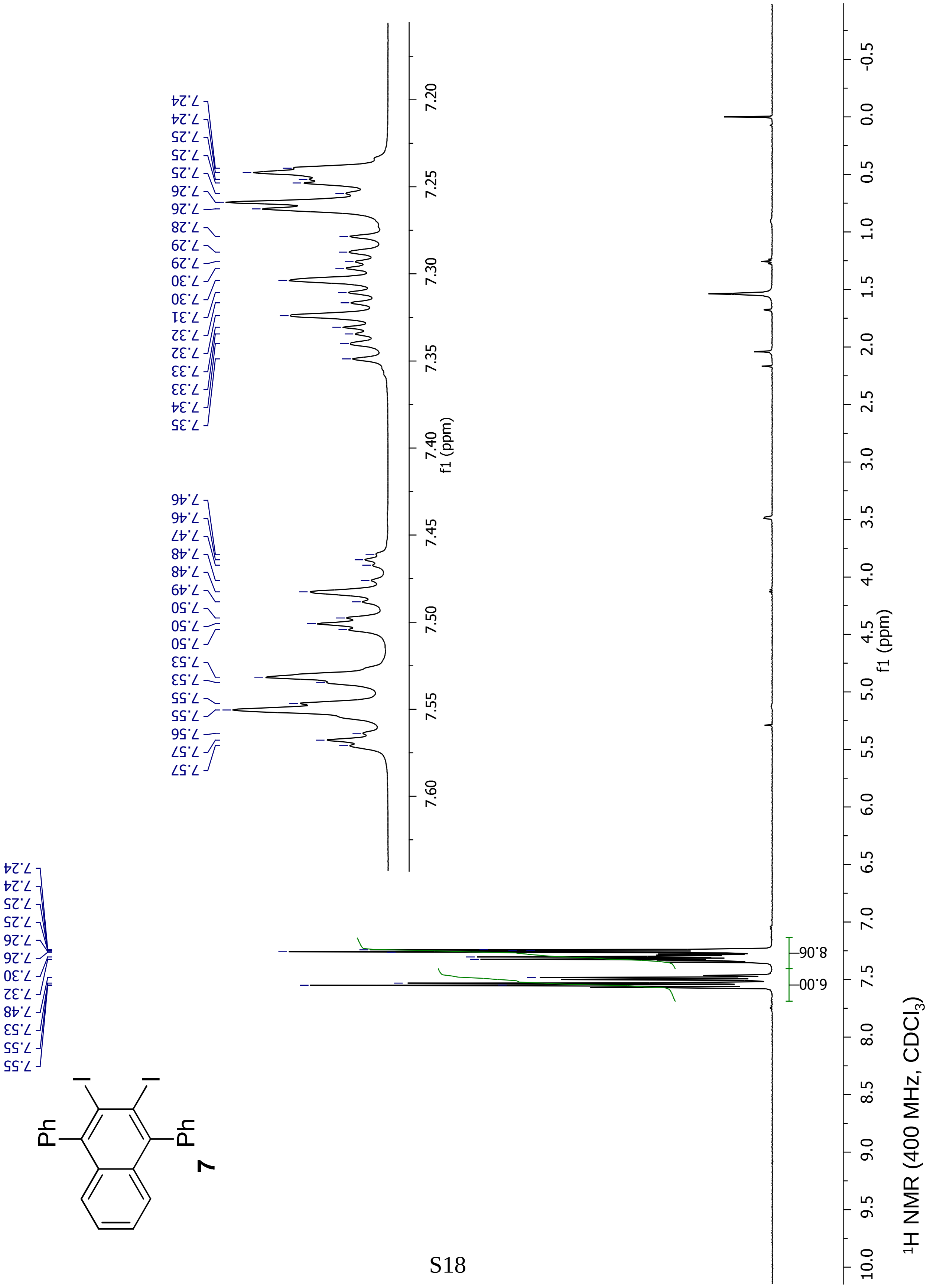

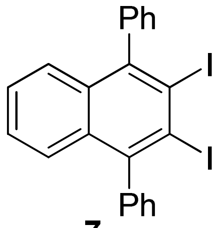

7

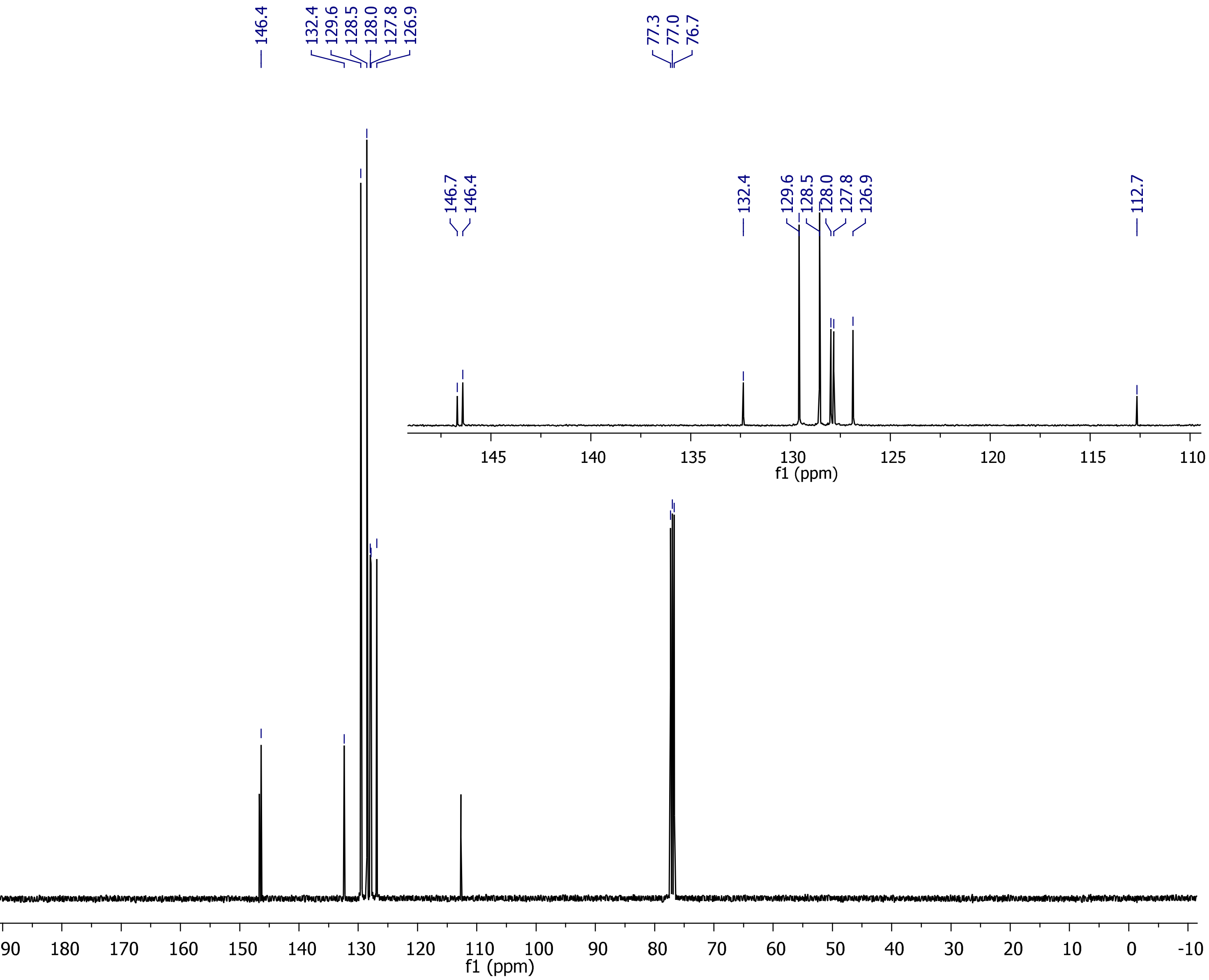

^{13}C NMR (100.6 MHz, $CDCl_3$)

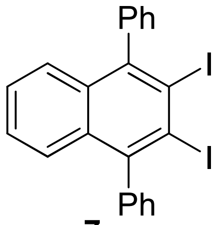
7

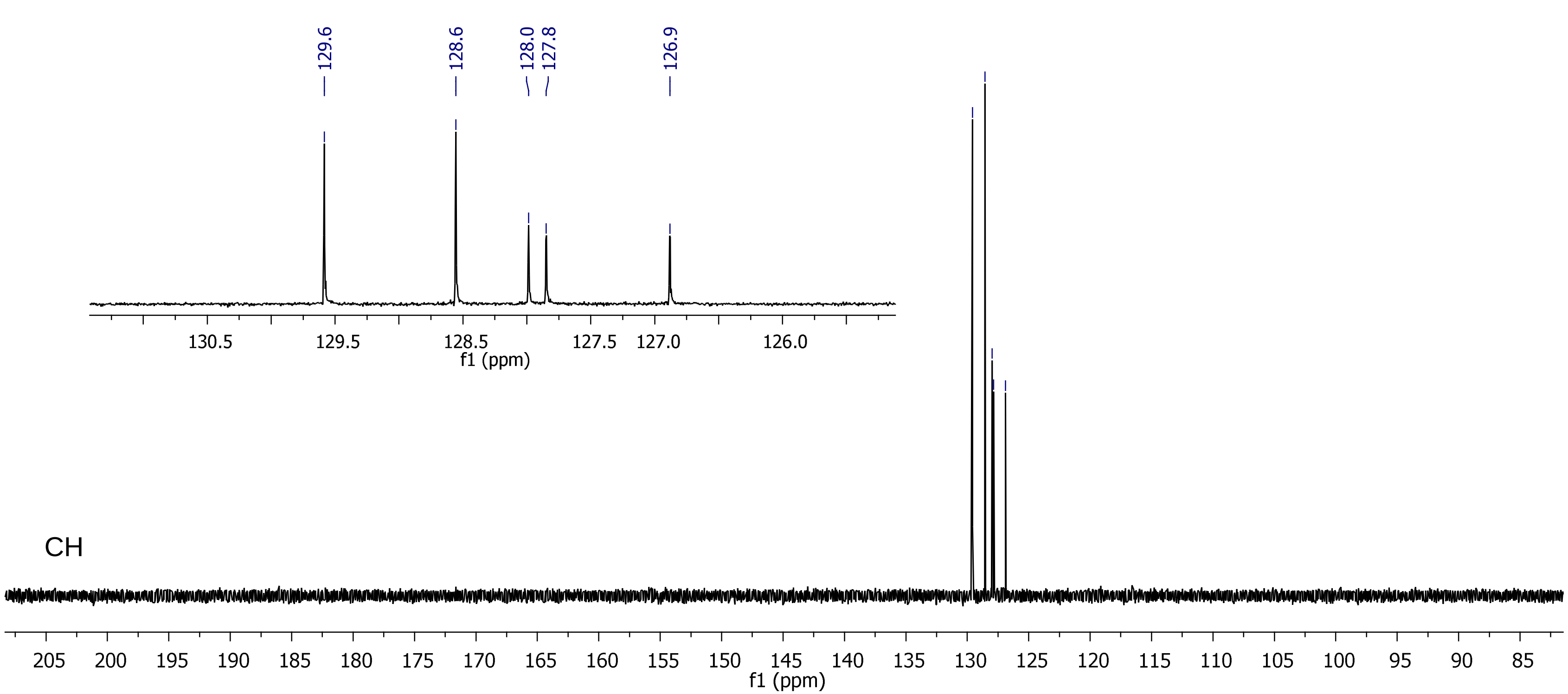

DEPT (100.6 MHz, $CDCl_3$)

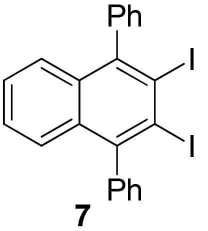

7

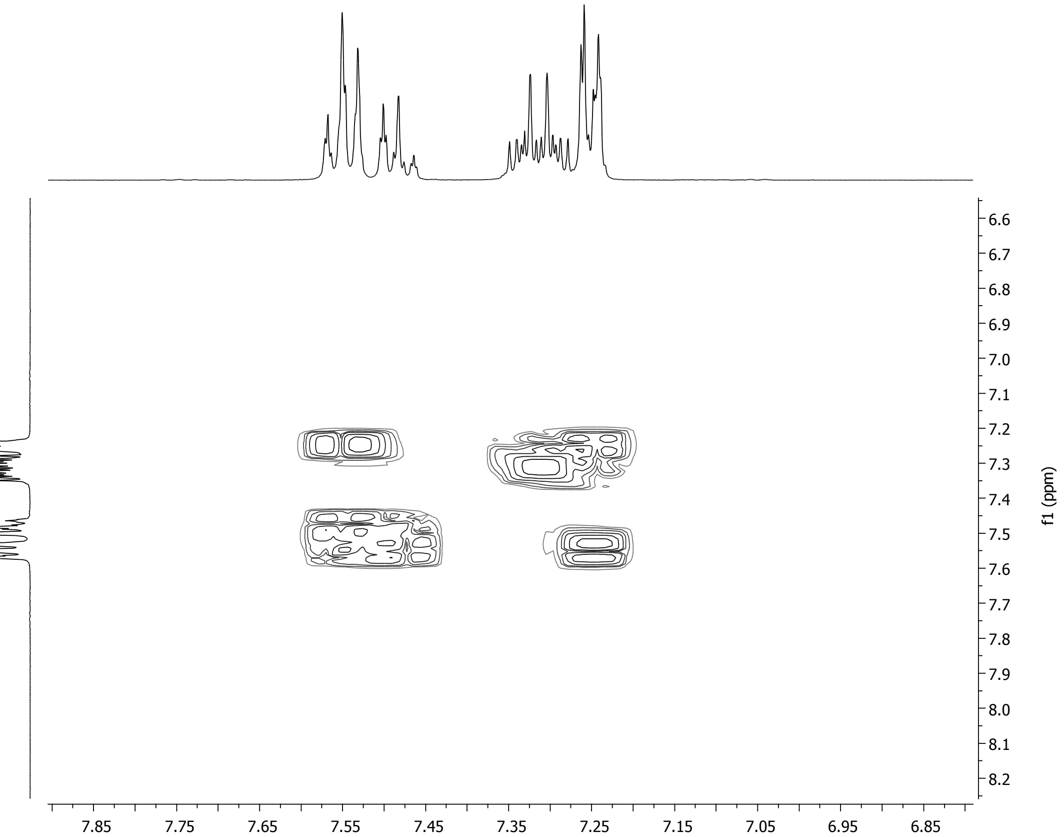

$^{1}H/^{1}H$ COSY (400 MHz, $CDCl_3$)

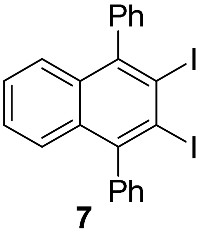

7

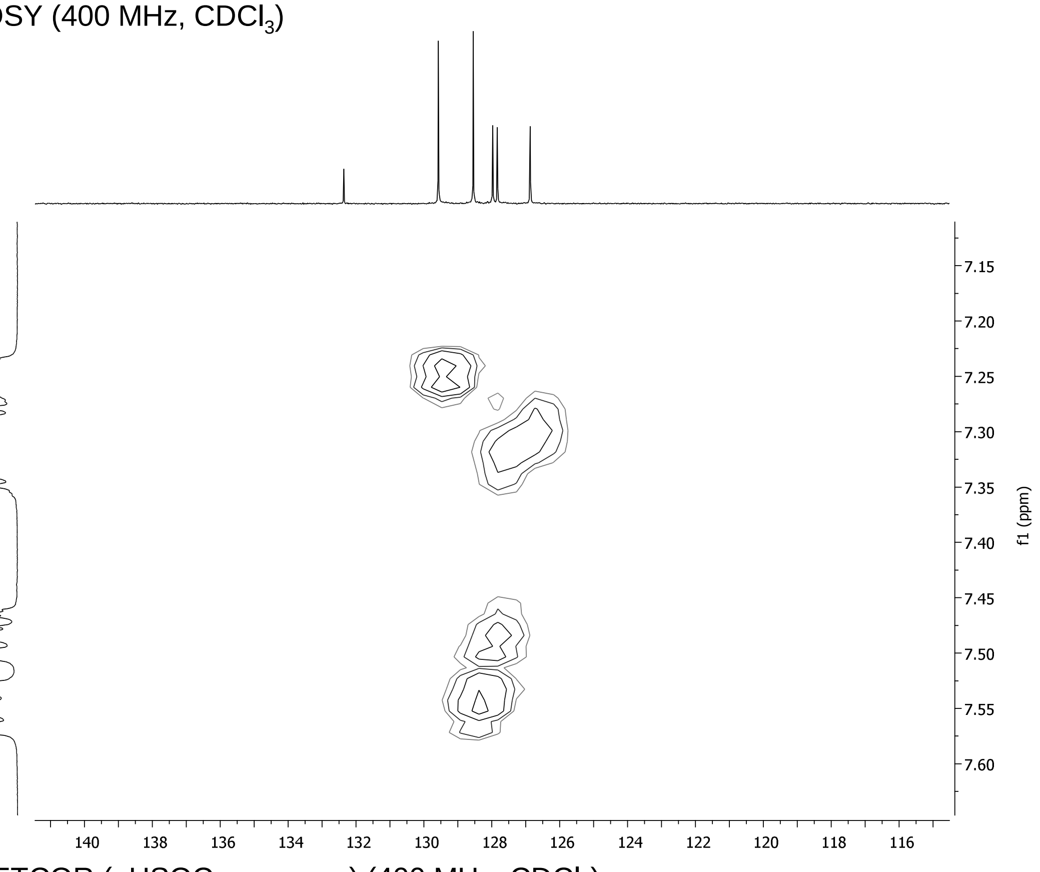

$^{1}H/^{13}C$ HETCOR (gHSQC sequence) (400 MHz, $CDCl_3$)

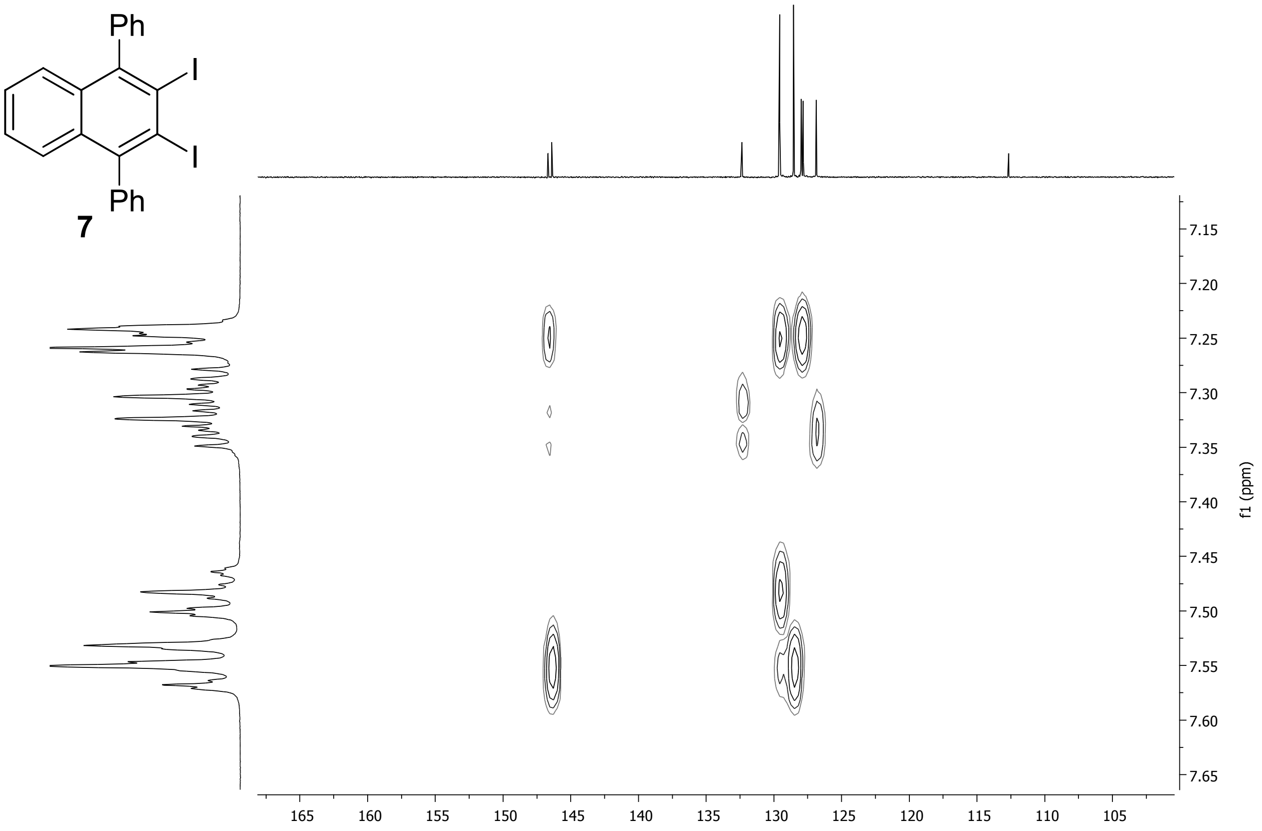

$^{1}H/^{13}C$ HETCOR (gHMBC sequence) (400 MHz, $CDCl_3$)

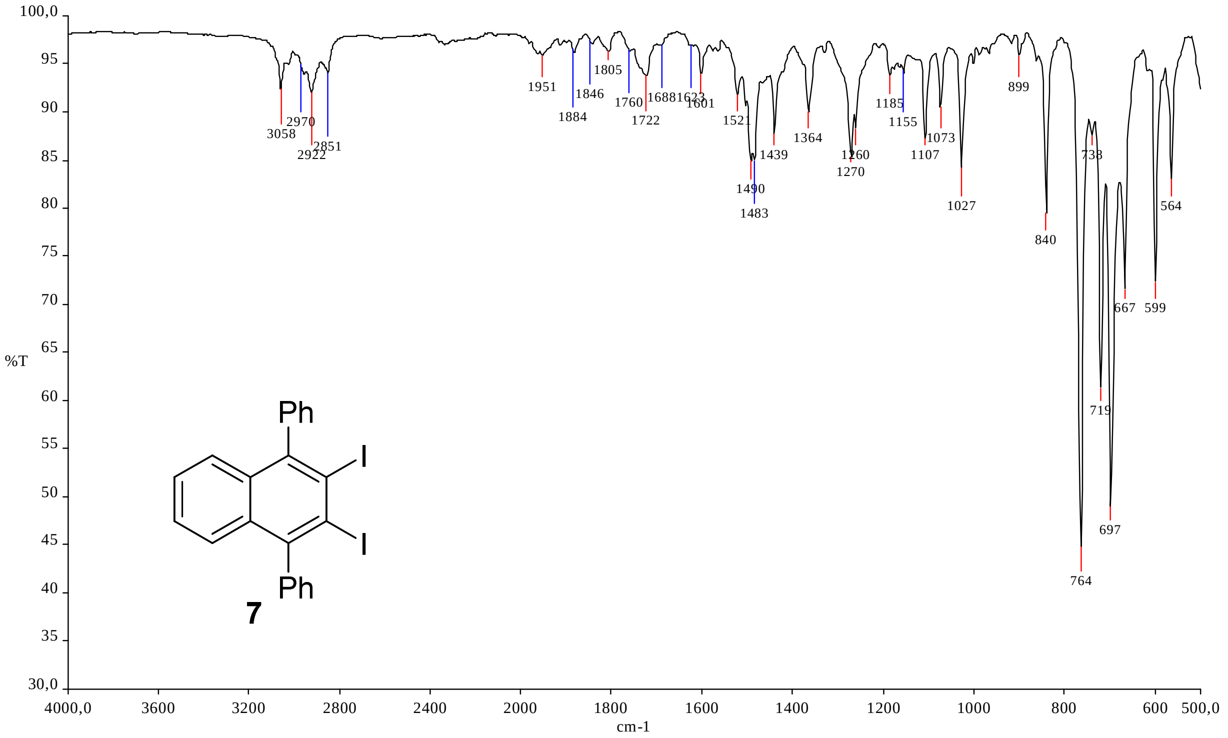

IR (ATR)
